# Protein electrostatic potential Fourier maps calculated using the transferable aspherical atom model and the independent atom model across resolutions

**DOI:** 10.1107/S2052252525008383

**Published:** 2025-10-17

**Authors:** Marta Kulik, Paulina Maria Dominiak

**Affiliations:** a University of Warsaw Faculty of Chemistry, Biological and Chemical Research Centre Żwirki i Wigury 101 Warszawa 02-089 Poland; University of California, Los Angeles, USA

**Keywords:** 3D electron diffraction, 3D ED, microcrystal electron diffraction, MicroED, 3D reconstruction and image processing, single-particle cryoEM, electron diffraction, multipolar scattering factors, electrostatic potential maps, resolution, transferable aspherical atom model (TAAM), micro-electron diffraction, cryogenic electron microscopy, macromolecular structure, proteins, quantum crystallography

## Abstract

Common sense tells us that experimental maps of lower (worse) resolution obtained from cryogenic electron microscopy or three-dimensional electron diffraction convey less information than maps of higher (better) resolution. However, information regarding the presence of charged moieties is more visible at lower resolutions. To investigate this phenomenon from a theoretical perspective, we analyzed the effects of truncation of data from the high-resolution end (from 1 Å to 8 Å) on theoretical Fourier images of the electrostatic potential of protein crystals, using both the popular independent atom model (IAM) of scattering factors and the more accurate transferable aspherical atom model (TAAM) combined with the UBDB/MATTS data bank. We compared our findings with those obtained for theoretical Fourier images of electron density maps associated with X-ray diffraction. Strikingly, when IAM is applied, there is almost no qualitative difference between the Fourier maps of electrostatic potential and electron density, regardless of their resolution. In contrast, the Fourier electrostatic potential maps calculated with TAAM, when of lower resolution, strongly differ from the electron density maps at the positions of charged moieties. Comparing TAAM and IAM, in the case of Fourier electrostatic potential maps, the relative difference between them is usually greatest at lower resolution maps, with a noticeable dependence on atom type and charge. In the case of Fourier electron density maps, this relative difference is much smaller and becomes more apparent in higher resolution maps. Thus, the use of accurate scattering factors is much more important for lower resolution data than for higher resolution data if one wants to investigate charged systems.

## Abstract 2

Electrostatic potential maps of proteins are calculated at various resolutions using the transferable aspherical atom model (TAAM) to determine the relations between the electrostatic potential and resolution for different atom types.

## 1. Introduction

To unravel the molecular mechanisms that govern the function and activity of living organisms, it is crucial to examine the three-dimensional structures of their constituent molecules, aiming for the highest possible level of reliability. This reliability hinges on the resolution of the obtained data, which varies in definition depending on the experimental technique employed. X-ray diffraction provides an indirect measurement of the electron density of the studied molecule, while the electrostatic potential of the molecule is assessed either indirectly through 3D electron diffraction (3D ED, MicroED) or directly via single-particle cryogenic electron microscopy (single-particle cryo-EM). In the field of X-ray and electron diffraction, resolution is strictly defined based on the maximum diffraction angles at which reflections can still be observed. In the field of single-particle cryo-EM, there is a general problem with the definition of resolution (Vilas *et al.*, 2022 ▸; Lander, 2024 ▸). A common but imperfect standard used to define the global resolution of experimental data is the Fourier shell correlation (FSC) method (Harauz & van Heel, 1986 ▸). FSC is based on the similarity of two half-maps, generated independently from two non-overlapping halves of the particles’ data set. These half-maps are then compared in Fourier space by band-pass filtering at subsequent resolution shells. The ‘gold-standard’ FSC threshold of 0.143 is widely accepted to define the resolution of the map (Rosenthal & Henderson, 2003 ▸). The resolutions reported by FSC-based methods should be treated with caution as they might be influenced by the presence of additional sources of correlation between two presumably independent data sets, such as systematic errors (Bromberg *et al.*, 2020 ▸; Chari & Stark, 2023 ▸). It has also been noted that FSC might be more suited to reflect reproducibility rather than resolution (Subramaniam *et al.*, 2016 ▸).

Despite the issues concerning the definition of resolution, it is undeniable that recent years have brought enormous progress in single-particle cryo-EM in terms of resolution and quality of the obtained experimental data of the 3D structures of macromolecules and macromolecular complexes (Namba & Makino, 2022 ▸). Many barriers have been overcome, allowing atomic level resolution to be reached for several model proteins, such as apoferritin (Yip *et al.*, 2020 ▸; Nakane *et al.*, 2020 ▸; Maki-Yonekura *et al.*, 2023 ▸; Küçükoğlu *et al.*, 2024 ▸). A significant advance has also been made in the field of understanding the theory behind the differences between the Fourier images of electrostatic potential and electron density (Bochtler, 2024 ▸). In contrast to electron density maps, which are shaped by the X-rays scattered by the negatively charged electron cloud, electrostatic potential maps are also significantly influenced by the positively charged atomic nuclei (Marques *et al.*, 2019 ▸). Moreover, the electron scattering that shapes the electrostatic potential maps is more efficient at revealing the subtle structural details compared with X-ray scattering due to the very short wavelength and larger scattering cross section (Bendersky & Gayle, 2001 ▸). For negatively charged atoms at low scattering angles the amplitudes of electron scattering factors become negative (Marques *et al.*, 2019 ▸; Jha *et al.*, 2021 ▸) and must be parametrized with great care (Yonekura & Maki-Yonekura, 2016 ▸). The influence of charges on experimental electrostatic potential maps has been demonstrated by 3D ED (Mitsuoka *et al.*, 1999 ▸; Yonekura *et al.*, 2015 ▸; Blum *et al.*, 2021 ▸) and single-particle cryo-EM (Radon *et al.*, 2020 ▸; Maki-Yonekura *et al.*, 2023 ▸; Bick *et al.*, 2024 ▸). Modeling the charged species using IAM in the refinement of metal–organic complexes against 3D ED data, especially when assigning formal charges based on the oxidation state of iron, does not accurately represent the experimental electrostatic potential map and more sophisticated scattering models are necessary (Pacoste *et al.*, 2024 ▸). Apart from scattering factor models, preferential radiation damage of Asp and Glu residues is also recognized as a significant factor that affects the observed densities of these residues in electrostatic potential maps (Bartesaghi *et al.*, 2014 ▸). Furthermore, electron diffraction measurements are influenced by dynamical scattering (Palatinus *et al.*, 2017 ▸; Blum *et al.*, 2021 ▸).

There are several different approaches to modelling the electron density of atoms (Kulik & Dominiak, 2022 ▸). Here, we take into account a traditional, simple and spherical model frequently applied in X-ray diffraction, called the independent atom model (IAM), and a more advanced, multipolar model – the transferable aspherical atom model (TAAM). TAAM is based on the idea of transferability of the multipolar parameters of the same atom types obtained in one chemical environment to a similar chemical environment, for example from a small organic molecule to a large macromolecular complex. TAAM can be used with various data banks containing sets of parameters specific for particular atom types. Here, we use the University at Buffalo Data Bank (UBDB) (Dominiak *et al.*, 2007 ▸; Jarzembska & Dominiak, 2012 ▸; Kumar *et al.*, 2019 ▸), which is currently being developed under the name the Multipolar Atom Types from Theory and Statistical clustering (MATTS) data bank (Jha *et al.*, 2022 ▸; Rybicka *et al.*, 2022 ▸) and is based on the Hansen–Coppens equation. TAAM combined with UBDB/MATTS allows charge transfer between atoms to be taken into account, as well as radial (spherical) and angular (aspherical) deformations of their electron densities due to formation of chemical bonds and other interatomic interactions within a molecule. As a result, this model describes the electron density in a more accurate way than IAM. It is reflected for example in the better quality of coordinates and thermal displacement parameters from TAAM refinements against X-ray diffraction data (Bąk *et al.*, 2011 ▸). Moreover, TAAM significantly improves the lengths of the *X*—hydrogen bonds, making them comparable to bond lengths observed in neutron diffraction (Jha *et al.*, 2020 ▸). So far, TAAM with UBDB/MATTS has been used in a wide range of studies on electron density of proteins and nucleic acids (Malińska *et al.*, 2014 ▸; Kulik *et al.*, 2015 ▸; Kumar & Dominiak, 2021 ▸). The comparison of electron density and electrostatic potential calculated for a series of organic molecules using TAAM and the more computationally demanding conventional quantum mechanics methods has demonstrated very good agreement in numerous studies (Volkov & Coppens, 2004 ▸; Volkov *et al.*, 2004 ▸; Jarzembska & Dominiak, 2012 ▸; Kumar *et al.*, 2014 ▸; Kumar *et al.*, 2019 ▸; Gruza *et al.*, 2020 ▸). TAAM has also been applied to electron diffraction on small-molecule crystals, using the Mott–Bethe equation, resulting in lower *R* factors and lower residuals in the Fourier difference map (Gruza *et al.*, 2020 ▸; Jha *et al.*, 2021 ▸; Pacoste *et al.*, 2024 ▸; Kumar *et al.*, 2024 ▸; Olech *et al.*, 2024 ▸).

Recently, we have developed a method to model the electrostatic potential of macromolecules using TAAM via structure-factor calculation and Fourier summation (Kulik *et al.*, 2022 ▸). Theoretical Fourier TAAM maps generated at the same resolution as the experimental 3D electron diffraction electrostatic potential maps [those of the lysozyme structure at 1.8 Å resolution, EMD-8217 (de la Cruz *et al.*, 2017 ▸), and of proteinase K at 1.75 Å resolution, EMD-8077 (Hattne *et al.*, 2016 ▸)] have shown better agreement with the experimental data than IAM maps (Kulik *et al.*, 2022 ▸). In this work, we focus on the features of theoretical Fourier TAAM maps of macromolecules – both from X-ray diffraction and electron diffraction – generated at various resolutions and their relation to the behavior of the atomic scattering factors at various scattering angles. Thus, we lay the groundwork for the comparison of these theoretically generated maps with experimental maps of different resolutions in the future.

## 2. Methods

The theoretical background necessary to understand the derivation of the X-ray scattering factors and electron scattering factors for both the IAM and TAAM has been described in detail in our previous publication (Kulik *et al.*, 2022 ▸). Therefore we will mention here only two equations that set the foundations of our method.

The Hansen–Coppens equation (Hansen & Coppens, 1978 ▸) models the electron density of an atom by dividing it into two spherical terms, representing the core and valence electrons, and the aspherical multipole expansion term for valence electrons: 
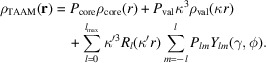
The spherical core ρ_core_ and spherical valence ρ_val_ terms are spherically averaged Slater-type functions, specific for isolated atoms of individual chemical elements, normalized to one electron, and multiplied by electron populations parameters *P*_core_ and *P*_val_. The κ parameter refers to the expansion or contraction of the spherical valence shell. The aspherical part is represented by the Slater-type radial functions (*R*_*l*_) together with a finite spherical harmonic expansion in a nucleus-centered local frame (*Y*_*lm*_). The population of multipole densities is represented by the *P*_*lm*_ parameter. The 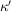 parameter reflects the expansion or contraction of the aspherical part.

The Mott–Bethe equation (Mott & Massey, 1965 ▸) is used to calculate the electron scattering factors *f*^e^ from the X-ray scattering factors *f*^X^: 
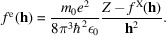
Here 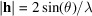, where θ denotes one half of the scattering angle and λ is the electron wavelength. *m*_0_, *e*, ℏ, ε_0_ and *Z* are the electron mass, electron charge, reduced Planck constant, the permittivity of vacuum and the atomic number, respectively.

To generate the Fourier maps of electrostatic potential and electron density at various resolutions, only the atomic coordinates, occupancies and *B* factors are necessary. To simulate electron diffraction data, we also took into account the experimental unit-cell parameters and space-group symmetry. We have used the same set of the atomic coordinates and *B* factors as in Kulik *et al.* (2022 ▸), namely the model of lysozyme structure from *Gallus gallus* at 1.8 Å resolution [PDB ID 5k7o (de la Cruz *et al.*, 2017 ▸)], and of proteinase K from *Parengyodontium album* at 1.75 Å resolution [PDB ID 5i9s (Hattne *et al.*, 2016 ▸)]. The models were downloaded from the RCSB PDB (Berman *et al.*, 2000 ▸). *Molprobity* (Williams *et al.*, 2018 ▸) and *Chimera* (Pettersen *et al.*, 2004 ▸) were used to add the hydrogen atoms to proteins and water molecules, respectively. The hydrogen atoms in proteins were adjusted to match the protonation state at pH 4.7 for lysozyme and pH 8 for proteinase K. The lengths of the covalent bonds to hydrogen atoms were corrected to the mean values from neutron diffraction (Allen & Bruno, 2010 ▸). The side chain of the partially missing residue Arg64 in proteinase K was rebuilt in *Maestro* 11.9 (Schrödinger Release 2019-1: Maestro, Schrödinger, LLC, New York, NY, 2019). The atomic *B* factors were assigned to hydrogen atoms according to 150% of the *B*-factor values of their closest non-hydrogen atoms for the methyl groups and water molecules and 120% for all other for protein hydrogen atoms (Lübben *et al.*, 2014 ▸). The same set of *B* factors was applied at every resolution. The *LSDB* program (Volkov *et al.*, 2004 ▸) was used to automatically assign the UBDB2018 (Kumar *et al.*, 2019 ▸) atom-type parameters [currently MATTS (Jha *et al.*, 2022 ▸; Rybicka *et al.*, 2022 ▸)] to all atoms. The multipole model parameters *P*_val_, *P*_*lm*_, κ and κ′ were manually adjusted for the S atoms from 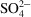 molecules and for water molecules 401, 408 and 480 in the proteinase K structure due to missing atom types or small distances between atoms that caused errors in the assignment process. The multipole parameters of the water molecule No. 480 were multiplied by ½ due to its location at the symmetry element.

The *Python* 3.7 script provided in Kulik *et al.* (2022 ▸) was applied to generate reflection indices for structure-factor calculations up to 1 Å resolution. The DiSCaMB library, available at https://4xeden.uw.edu.pl/software/ (Chodkiewicz *et al.*, 2018 ▸), was used to calculate the structure factors for X-ray diffraction for the TAAM with UBDB2018/MATTS parameters at the given resolution and then to convert them to the structure factors for electron diffraction using the Mott–Bethe equation. The X-ray (Doyle & Turner, 1968 ▸; Fox *et al.*, 1989 ▸) and electron (Peng *et al.*, 1996 ▸) scattering-factor coefficients for the IAM were taken directly from *International Tables for Crystallography* (2006 ▸), Vol. C, Tables 6.1.1.4 and 4.3.2.3. The 3D Fourier maps of electrostatic potential (denoted as eTAAM and eIAM) were generated with TAAM and IAM electron scattering factors, and electron density 3D Fourier maps (xTAAM and xIAM) were generated with TAAM and IAM X-ray scattering factors using the *WinXD2016* package (Volkov *et al.*, 2016 ▸) *XDFOUR* module. The unit of electrostatic potential was recalculated to e Å^−1^, as discussed by Kulik *et al.* (2022 ▸). All 3D Fourier maps were generated with high-resolution cutoffs from 1 Å to 8 Å every 0.5 Å, and with voxel size 0.3 Å. The maps were generated in two versions: with or without taking into account the thermal smearing effects (denoted as ‘with *B*’ or ‘without *B*’, respectively). Additionally, four 3D Fourier maps for eTAAM and eIAM of lysozyme, taking into account the thermal smearing effects, were calculated using the high-resolution cutoffs 1 Å and 4 Å with the low-resolution cutoff set at 8 Å. To visualize the maps in *Chimera* (Pettersen *et al.*, 2004 ▸), the .grd map format was converted to situs format. The calculated 3D maps were trimmed with a 3 Å margin around the protein. For visualization, the 3D maps were scaled to match the standard deviation of the voxel values of the experimental density maps [0.162 and 0.166 for lysozyme, EMD-8217 (de la Cruz *et al.*, 2017 ▸) and proteinase K, EMD-8077 (Hattne *et al.*, 2016 ▸), respectively] in order to compare the data with our previous work (Kulik *et al.*, 2022 ▸). The mean voxel value was shifted to zero. The *Chimera* software (Pettersen *et al.*, 2004 ▸) was utilized for trimming, scaling, and the visualization of sigma contours in 3D. 2D Fourier eTAAM, eIAM, eTAAM − eIAM, xTAAM, xIAM and xTAAM − xIAM maps were calculated in the *WinXD2016* package (Volkov *et al.*, 2016 ▸) *XDFOUR* module using a 0.1 Å grid step. The 2D maps were not further scaled.

To quantitatively compare the Fourier maps calculated at resolutions from 1 Å to 8 Å sampled every 0.5 Å, unscaled 3D Fourier maps were used. The amount of electrostatic potential or electron density associated with selected types of atoms in the studied structures was quantified by computing the average value of the map in the volume surrounding the atom with the covalent radius averaging method, as described by Kulik *et al.* (2022 ▸). The method takes into account the 3D grid-point values sampled within the volume up to the covalent radius distance from atom positions. As a result, each atom has its individual covalent radius value, number of grid points and a sum of grid-point values. The sum of grid-point values was divided by the number of grid points to obtain the average value of the grid point specific for the given atom. Next, the atoms belonging to the same atom type were collected and the average value of average values specific for an individual atom was computed as detailed in Section S4 in Kulik *et al.* (2022 ▸). Thus, there was no need to align the molecules within this procedure. Every unscaled 3D Fourier electrostatic potential and electron density TAAM map, generated with a 0.3 Å grid, was sampled in *Chimera* using a 0.1 Å grid. In addition to the average value, the integrated value of the map for the volume surrounding the atom was computed by multiplication of the average value by the number of grid points, listed in Table S1, and normalizing the value to correspond to sampling of one per Å^3^.

To visualize the dependence of the atomic scattering factors on scattering angle, the atomic scattering factors for selected atom types were calculated using the *DiSCaMB* library for atom types from the UBDB/MATTS data bank. Only spherical core and spherical valence contributions, as defined in the Hansen–Coppens equation, were included in the calculations. The list of selected atom types with their *P*_core_ and *P*_val_ values is given in Table S2. For comparison, the atomic scattering factors for isolated atoms were added to the analysis. In the case of isolated atoms, the κ parameter was set to 1 and the population of valence electrons, *P*_val_, was set to the number of valence electrons in a neutral atom or an isolated ion (Table S2). First, the X-ray atomic scattering factors were calculated, with 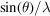 sampled every 0.0025 in the range 0.0000–0.0250, every 0.0125 in the range 0.0250–0.0750, and every 0.0250 in the range 0.0750–0.5000. Secondly, they were transformed to the electron scattering factors with the Mott–Bethe equation, using the multiplier equal to 0.023934 specified by Peng (1999 ▸). The atomic scattering factors that take into account the thermal smearing effects were calculated by multiplying the atomic scattering factors by the temperature factor 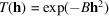, where *B* is the average *B* factor measured for each atom type in the experimentally determined structures and listed in Table S3.

In order to compare our theoretical results with experimental data, we have also constructed a structural model of lysozyme containing an additional 762 water molecules to fill the whole unit-cell volume. The water oxygen atoms were added to the asymmetric unit in *Coot* (Emsley *et al.*, 2010 ▸), with automatic assignment of the *B* factors. The mean *B* factor was 39 Å^2^ and the values ranged from 10 Å^2^ (assigned to 20 oxygen atoms) up to 80 Å^2^ (assigned to 203 oxygen atoms). The water molecules were protonated, and their energy minimization was subsequently performed in *Chimera*. The *B*-factor values for hydrogen atoms were assigned as 150% of the corresponding oxygen-atom values. To mimic ‘bulk solvent’, the *B* factors of the newly added water oxygen atoms were increased by 300% relative to the values originally assigned by *Coot*. The original model of lysozyme deposited in the RCSB PDB contains one Na^+^ and two Cl^−^ ions, and the net charge of the system is +8 e. In order to neutralize the system and avoid adding counterions at ambiguously defined positions, we scaled the net charge of all water molecules in the system to −8 e instead of zero. The 3D Fourier maps were generated with high-resolution cutoffs of 1 Å and 4 Å, and additional maps were also generated with the low-resolution cutoff set at 8 Å. Wilson plots were derived by averaging the squared structure factors within resolution shells defined by 0.01 Å^2^ increments of reciprocal squared resolution (1/*d*^2^). The mean deviation between the squared experimental and theoretical structure factors, computed across intervals determined by the Wilson plot in the range 0.01 ≤ 1/*d*^2^ ≤ 0.31, served as a scale factor.

Chosen 3D Fourier maps of electrostatic potential and electron density, structure factors, and the associated structural models for lysozyme and proteinase K are available online in the Repository for Open Data (RepOD, Inter­disciplinary Centre for Mathematical and Computational Modelling, University of Warsaw, Warsaw, Poland, https://repod.icm.edu.pl/) using the doi https://doi.org/10.18150/7NQCCU for the lysozyme dataset (Kulik & Dominiak, 2025*a* ▸), https://doi.org/10.18150/K4A12G for the protein­ase K dataset (Kulik & Dominiak, 2025*b* ▸) and https://doi.org/10.18150/QJ6U6S for the lysozyme with bulk solvent dataset (Kulik & Dominiak, 2025*c* ▸). Deposited structure-factor files contain the reflection indices up to 1 Å resolution and two sets of Fourier coefficients, corresponding to the real and imaginary parts of the TAAM and IAM structure factors. The structure-factor files make it possible to generate the 3D Fourier maps of electrostatic potential and electron density of the studied proteins calculated with TAAM or IAM, with or without taking into account the thermal smearing effects, at any given resolution worse than or equal to 1 Å.

## 3. Results

**Figure 1:**
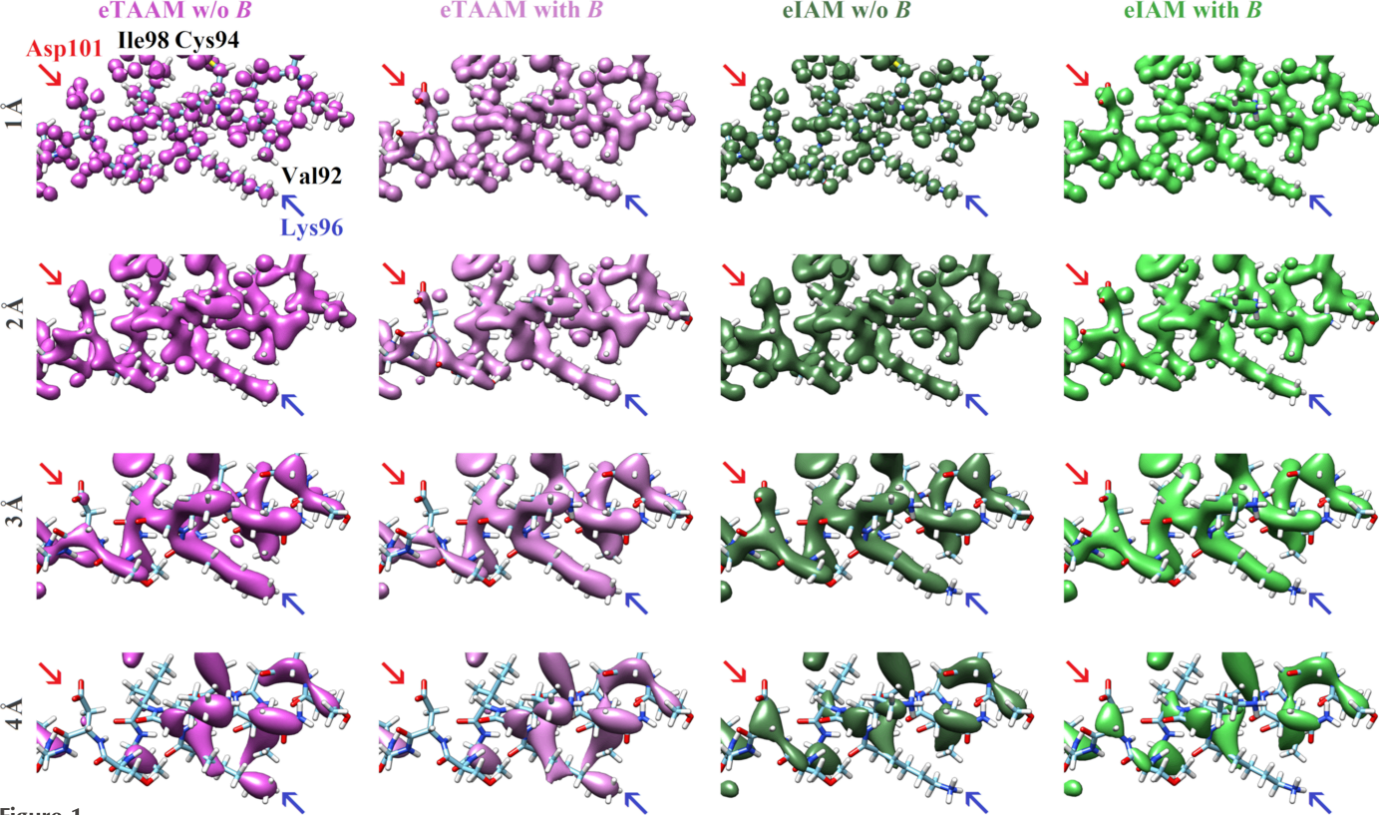
3D Fourier electrostatic potential maps of lysozyme at resolutions *d*_min_ = 1–4 Å, with an atomic structure shown only for a short helical fragment. The maps are calculated using TAAM without and with accounting for *B* factors, *i.e.* thermal smearing effects (magenta and pink, respectively) and using IAM without and with *B* factors (dark green and light green, respectively). Two chosen, oppositely charged amino acids are indicated with arrows. All maps are shown at the 2σ contour level.

Fourier maps of electrostatic potential have been modeled at different resolutions using the TAAM and IAM electron scattering factors, and the resulting 3D Fourier maps of a protein fragment are depicted in Fig. 1 ▸. The left panel shows the maps calculated without the influence of thermal smearing effects associated with experimental *B*-factor values (w/o *B*). Even though these maps are not physically meaningful, they allow investigation of the pure effect of truncation of the Fourier summation up to a certain resolution (a specific scattering angle). For maps at 1 Å resolution, the differences between the electrostatic potential maps based on the eTAAM and eIAM are difficult to notice. However, as the resolution worsens, several differences emerge at the positions of the charged amino acids. For the negatively charged Asp101, the eTAAM map contour encompasses less volume of positive electrostatic potential around carboxylate oxygen atoms at lower (worse) resolution than eIAM. It is visible that at 4 Å resolution a wide area around Asp101 is deprived of any positive electrostatic potential. An opposite trend is found for the positively charged Lys96: at lower resolution the eTAAM map contour encompasses more volume than eIAM and the positive electrostatic potential of the ammonium group is completely missing in the eIAM map at 4 Å resolution.

In the second variant of the eTAAM and eIAM maps, shown in the right panel in Fig. 1 ▸, thermal smearing effects were included in the calculations (with *B*). Thermal smearing effects impact the 3D Fourier maps to a larger extent at high (better) resolution than at lower (worse) resolution. The differences between eTAAM and eIAM maps at the positions of charged amino acids are already revealed at 1 Å resolution. As the resolution gets lower, smaller differences are visible between the maps calculated without and with *B* factors.

Contrary to the charged residues, no easy-to-notice differences between eTAAM and eIAM maps were observed for neutral amino-acid side chains. For example, the non-polar side chains of Ile98 and Val92 were deprived of positive electrostatic potential higher than the 2σ contour at 4 Å resolution both in eTAAM and eIAM maps. At the same time, the relatively heavy sulfhydryl group of Cys94 generated electrostatic potential higher than 2σ in all maps at 4 Å resolution. Also, a positive electrostatic potential from an aromatic ring from a neighboring amino acid close to Ile98 was present in all the maps and appears to have the same shape for eTAAM and eIAM maps at each resolution.

The importance of the contribution of reflections in the low-resolution range can be demonstrated by generating Fourier maps with the application of a low-resolution cutoff. We tested a low-resolution cutoff set at 8 Å, following the cutoff value applied in protein refinement against the electron diffraction data in Yonekura *et al.* (2015 ▸). The results are shown in Fig. S1. Filtering out low-resolution reflections clearly minimizes the discrepancies observed between eTAAM and eIAM, both at the resolution ranges 8–1 Å and 8–4 Å. This effect is especially visible for Asp101 in the theoretical maps calculated using the data from the resolution range 8–4 Å.

**Figure 2:**
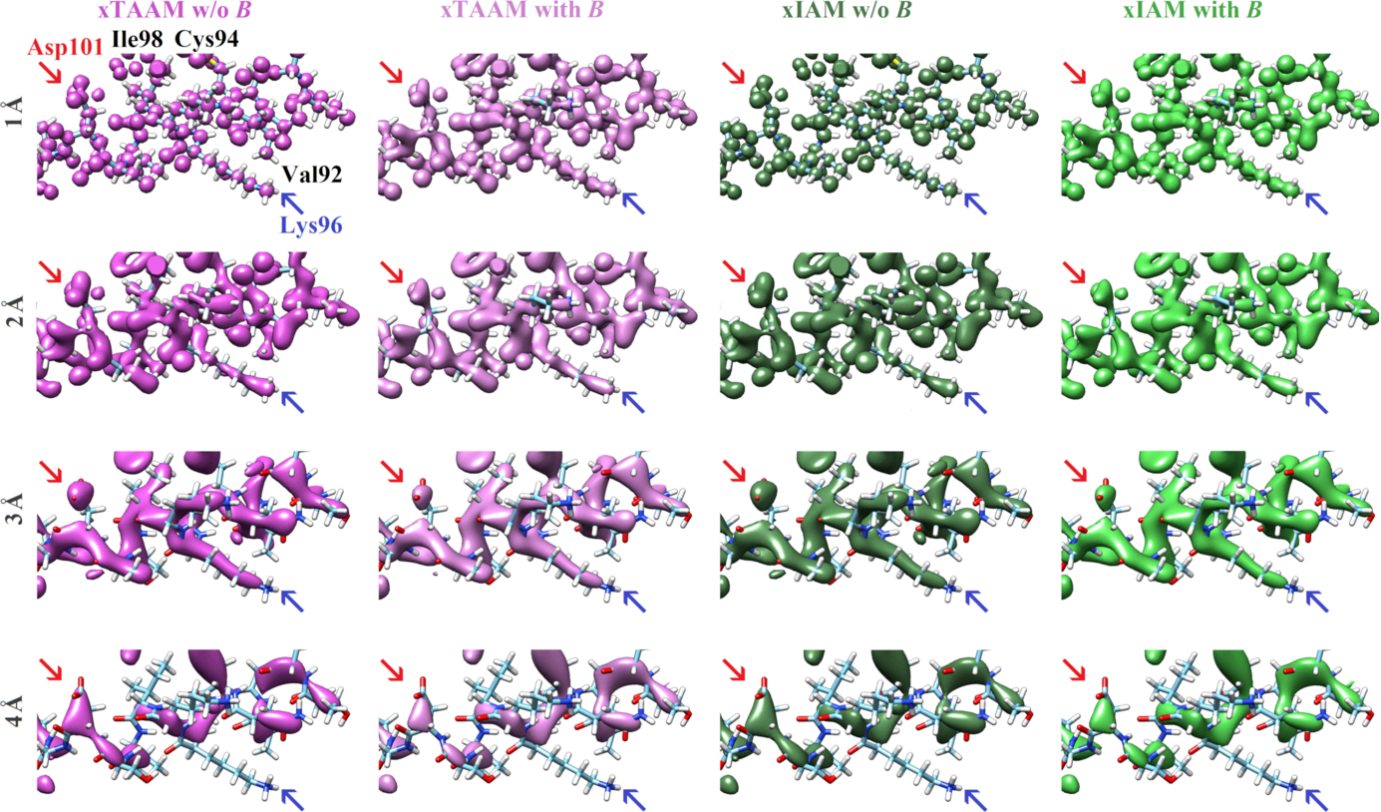
3D Fourier electron density maps of lysozyme at resolutions *d*_min_ = 1–4 Å, with an atomic structure shown only for a short helical fragment. The maps are calculated using TAAM without and with accounting for *B* factors, *i.e.* thermal smearing effects (magenta and pink, respectively) and using IAM without and with *B* factors (dark green and light green, respectively). Two chosen, oppositely charged amino acids are indicated with arrows. All maps are shown at the 2σ contour level.

In contrast to the eTAAM and eIAM maps, the differences between TAAM and IAM maps of electron density calculated with X-ray scattering factors (xTAAM and xIAM) at each resolution are miniscule (Fig. 2 ▸). If we compare them with the electrostatic potential maps, they strongly resemble eIAM maps – at low resolutions the density around the positively charged Lys96 is also flattened out, but the density close to the negatively charged Asp101 is still present. Similarly to in electrostatic potential maps, the impact of thermal smearing effects on the electron density maps is more pronounced at 1 Å resolution than at lower resolutions.

**Figure 3:**
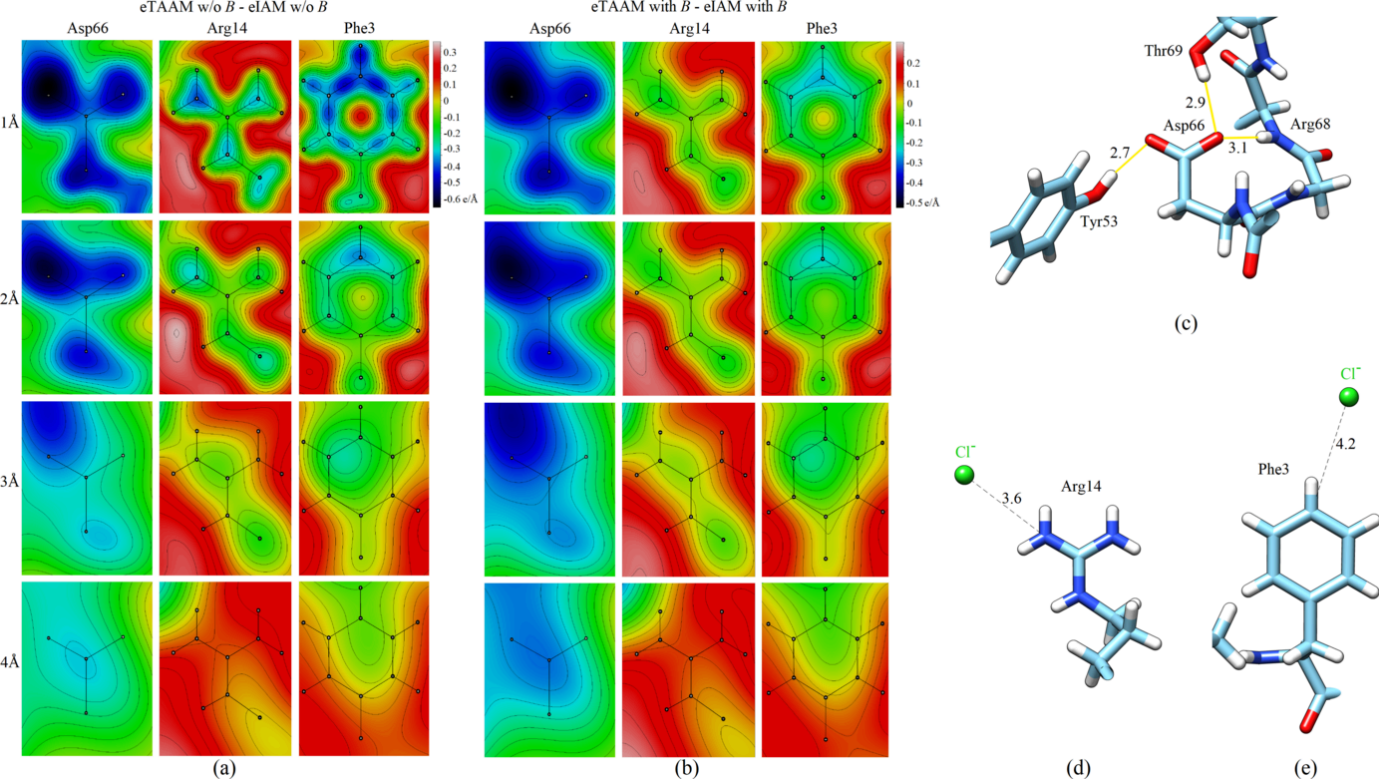
The 2D Fourier TAAM − IAM electrostatic potential maps of chosen lysozyme amino-acid side chains at resolutions *d*_min_ = 1–4 Å: (*a*) calculated without the thermal smearing effects (w/o *B*) and (*b*) calculated with the thermal smearing effects (with *B*). (*c*) Hydrogen-bond network around Asp66 marked as yellow lines. (*d*) Chloride ion close to Arg14. (*e*) Chloride ion close to Phe3. All distances are measured between two closest non-hydrogen atoms and are shown in Å.

In order to capture the tiny differences between various maps at positions of single amino-acid residues, the 2D Fourier TAAM − IAM difference maps (deformation maps) were investigated. Fig. 3 ▸(*a*, *b*) depicts sections through the planes of the carboxylate group of Asp66, the guanidinium group in Arg14 and the aromatic ring in Phe3. The eTAAM − eIAM map of Asp66 without thermal smearing effects reveals negative values, with the minimum value reaching −0.63 e Å^−1^ at 1 Å resolution (Table S4) located near the left-hand oxygen atom. This stems from the fact that the eIAM map contains more positive values in this region than eTAAM, as shown in Fig. S2. Thus, if the relative change between eTAAM and eIAM is calculated with respect to eTAAM at the left-hand oxygen-atom position, it will be negative. It is noteworthy that the worse the resolution, the larger the relative difference between eTAAM and eIAM is. For example, at 1 Å resolution it reaches −6% for the Asp66 map, while at 4 Å resolution it is −80% (Table S4). The presence of thermal smearing [Fig. 3 ▸(*b*)] results in flattened extrema, with the minimum value near the left-hand oxygen atom equal to −0.51 e Å^−1^ at 1 Å resolution. Nevertheless, the trend in the relative change between eTAAM and eIAM with thermal smearing is similar (Table S4 and Fig. S3).

An asymmetry in the electrostatic potential distribution around the left-hand oxygen atom of the carboxylate group, which is also visible at worse resolutions, is caused mainly by the imbalanced charge compensation from the surrounding hydrogen-bond donors, illustrated in Fig. 3 ▸(*c*). The maps calculated for an isolated Asp66 residue support the hypothesis that the hydrogen-bond network is responsible for the elongated electrostatic potential shape in this region (see Fig. S4, right panel). This shape is not affected by an Na^+^ ion, located 4.9 Å from the right-hand oxygen atom, as demonstrated by the calculations done for lysozyme deprived of this Na^+^ ion (Fig. S4). The presence of the Na^+^ ion has a moderate impact on the depth of the minima at oxygen-atom positions in 2D Fourier TAAM − IAM difference maps, while it does not affect the overall electrostatic potential shape. Interestingly, in the maps devoid of the Na^+^ ion, the relative change between eTAAM and eIAM with respect to eTAAM at the left-hand oxygen-atom position at 4 Å resolution was equal to −153% for Asp66 in the protein environment and −122% for the isolated Asp66 (see Table S5).

The 2D Fourier TAAM − IAM difference map of the positively charged Arg14 side chain is influenced by a chloride anion [Fig. 3 ▸(*d*)]. At 1 Å resolution, without the thermal smearing effect, the area of the negative electrostatic potential around the positions of the nitrogen atoms extends towards the positions of hydrogen atoms. The surrounding deformation electrostatic potential is positive. At lower resolutions or with thermal smearing, this extension of the negative electrostatic potential towards the positions of hydrogen atoms is not present. The difference map no longer shows negative values at nitrogen-atom positions at 4 Å resolution, but the negative electrostatic potential of the chloride ion is still observed. The same chloride ion is present in the vicinity of Phe3 [Fig. 3 ▸(*e*)] and its influence on the deformation density is also clearly visible, especially at lower resolutions. Note that this effect is hardly visible in the individual eTAAM and eIAM maps in Fig. S3. The deformation electrostatic potential map of Phe3 at 1 Å resolution shows the very essence of the multipolar model: strong negative signals are visible at the covalent bonds which underline the aspherical character of TAAM.

**Figure 4:**
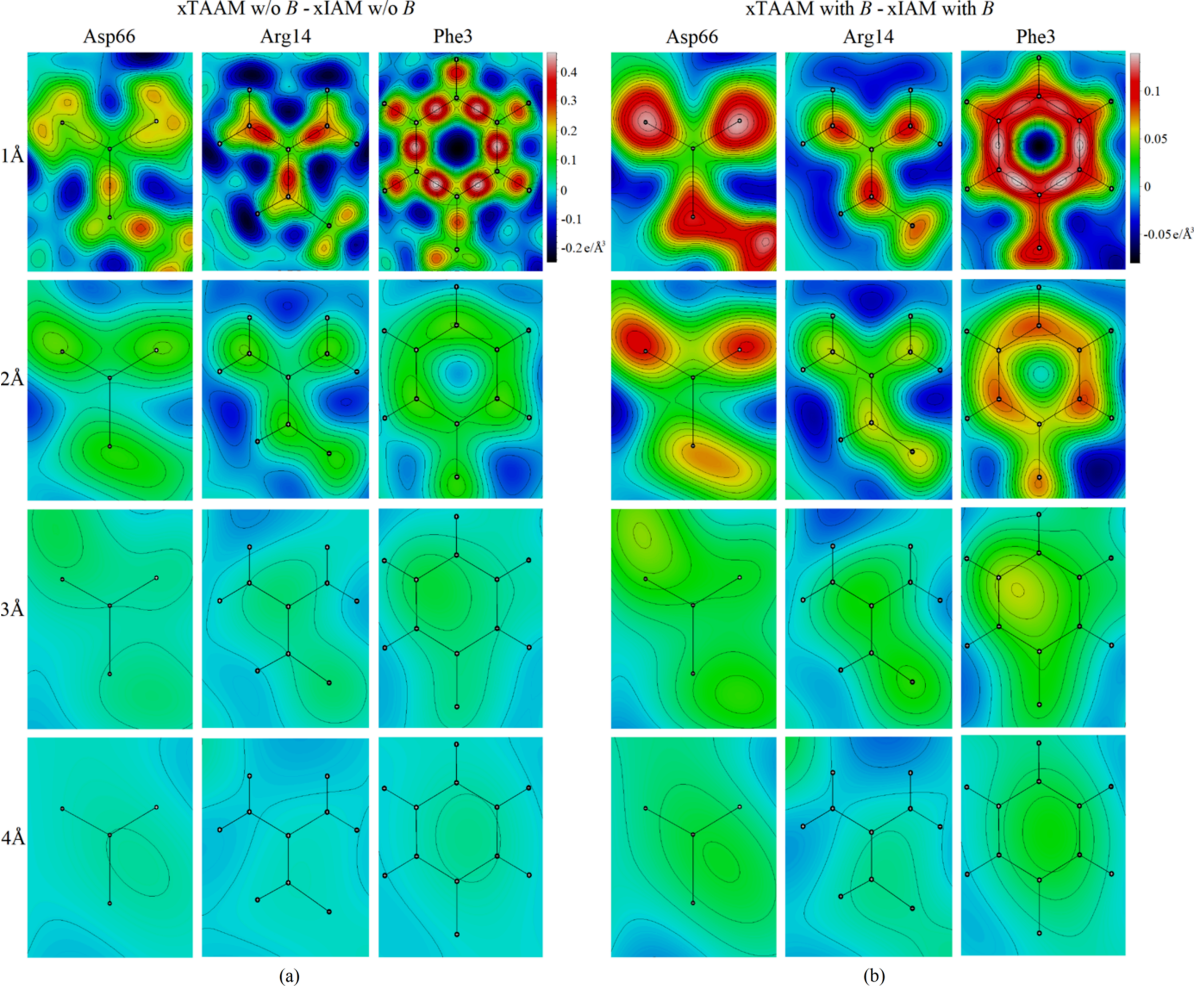
The 2D Fourier TAAM − IAM electron density maps of chosen lysozyme amino-acid side chains at resolutions *d*_min_ = 1–4 Å: (*a*) calculated without the thermal smearing effects (w/o *B*) and (*b*) calculated with the thermal smearing effects (with *B*).

The presence of positive peaks at the covalent bonds and at the lone electron pairs of oxygen is also spectacular in the 2D Fourier TAAM − IAM maps for X-ray diffraction at 1 Å resolution, the best resolution studied here [Fig. 4 ▸(*a*)]. For Phe3 [Fig. 4 ▸(*b*)], the peaks at the covalent bonds remain even if thermal smearing is taken into account. However, neither the xTAAM − xIAM maps nor the individual xTAAM and xIAM maps (Figs. S5 and S6) capture the effect of the compensation of the charge from the hydrogen bonds in Asp66 or the impact of the chloride ion in Arg14 and Phe3. Also, the relative change between xTAAM and xIAM with respect to xTAAM at the left-hand oxygen-atom position in Asp66 is not larger than 4% at all resolutions (Table S6).

**Figure 5:**
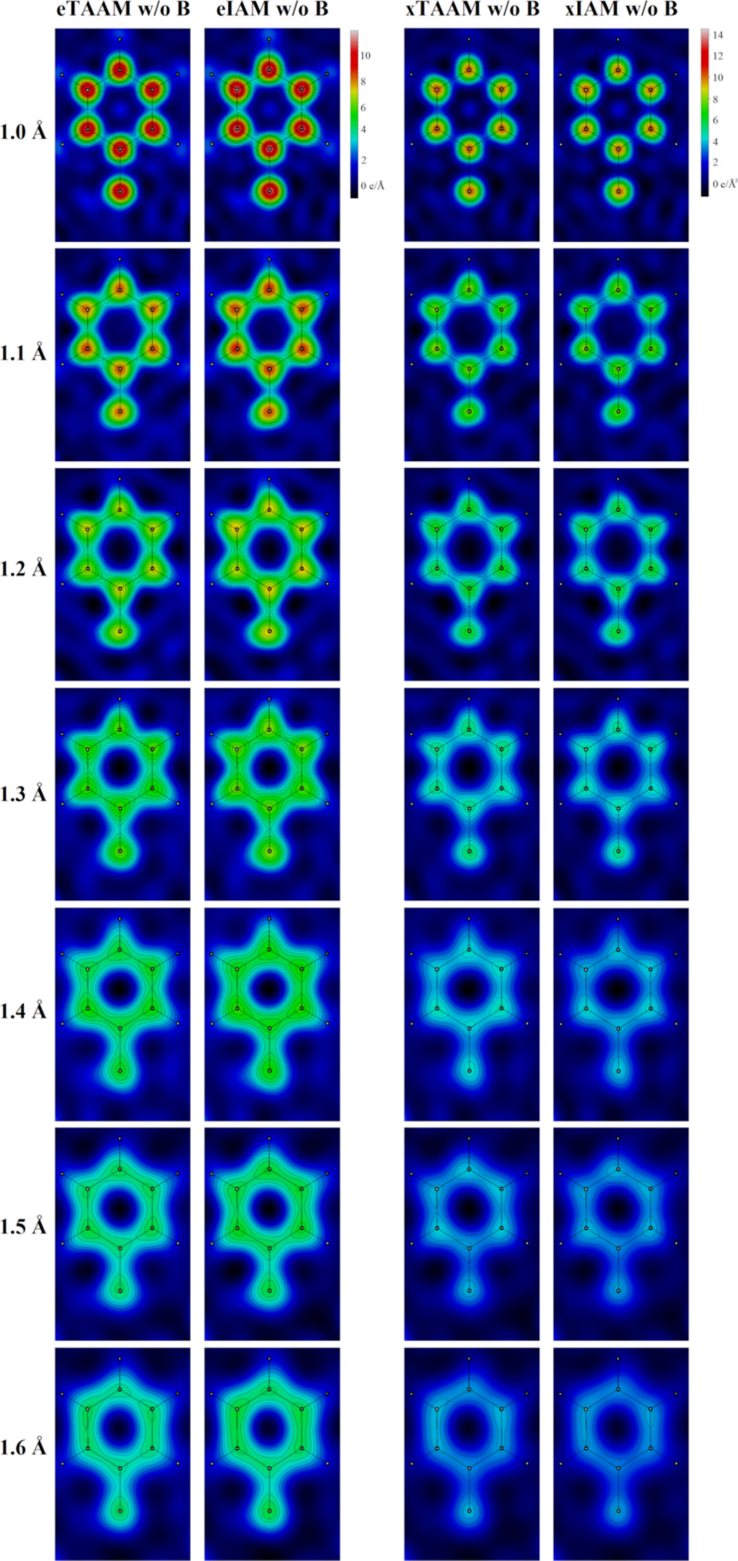
The 2D Fourier electrostatic potential density maps (eTAAM and eIAM) and electron density maps (xTAAM and xIAM) of the Phe3 side chain in lysozyme at resolutions *d*_min_ = 1.0–1.6 Å, calculated without the thermal smearing effects (w/o *B*).

The visibility of hydrogen atoms in the electrostatic potential and electron density maps was investigated by looking at the 2D Fourier TAAM and IAM maps of the aromatic ring plane in Phe3, see Fig. 5 ▸. Clear, distinct peaks in the vicinity of hydrogen atoms are revealed in the electrostatic potential maps generated without the thermal smearing effects at resolutions 1.0 and 1.1 Å and are barely visible even at 1.0 Å resolution in the electron density maps. The choice of TAAM brings a small but recognizable improvement in the ability to precisely locate the hydrogen atoms in the electrostatic potential maps, as the peaks appear to be slightly sharper. Note that neither of the maps of Phe3 calculated with the thermal smearing effects (Figs. S3 and S6) shows the separate hydrogen-atom peaks. As for the resolvability of carbon atoms, no difference between TAAM and IAM was observed. At 1.4 Å resolution in electrostatic potential maps and at 1.3 Å resolution in electron density maps, one can discern single peaks centered on the equivalent carbon atoms that form a symmetric aromatic ring. Respectively at 1.5 Å and 1.4 Å resolution, these peaks visibly lose their symmetry with respect to the center of the ring, while at lower resolutions, they become blurred and fused with each other.

**Figure 6:**
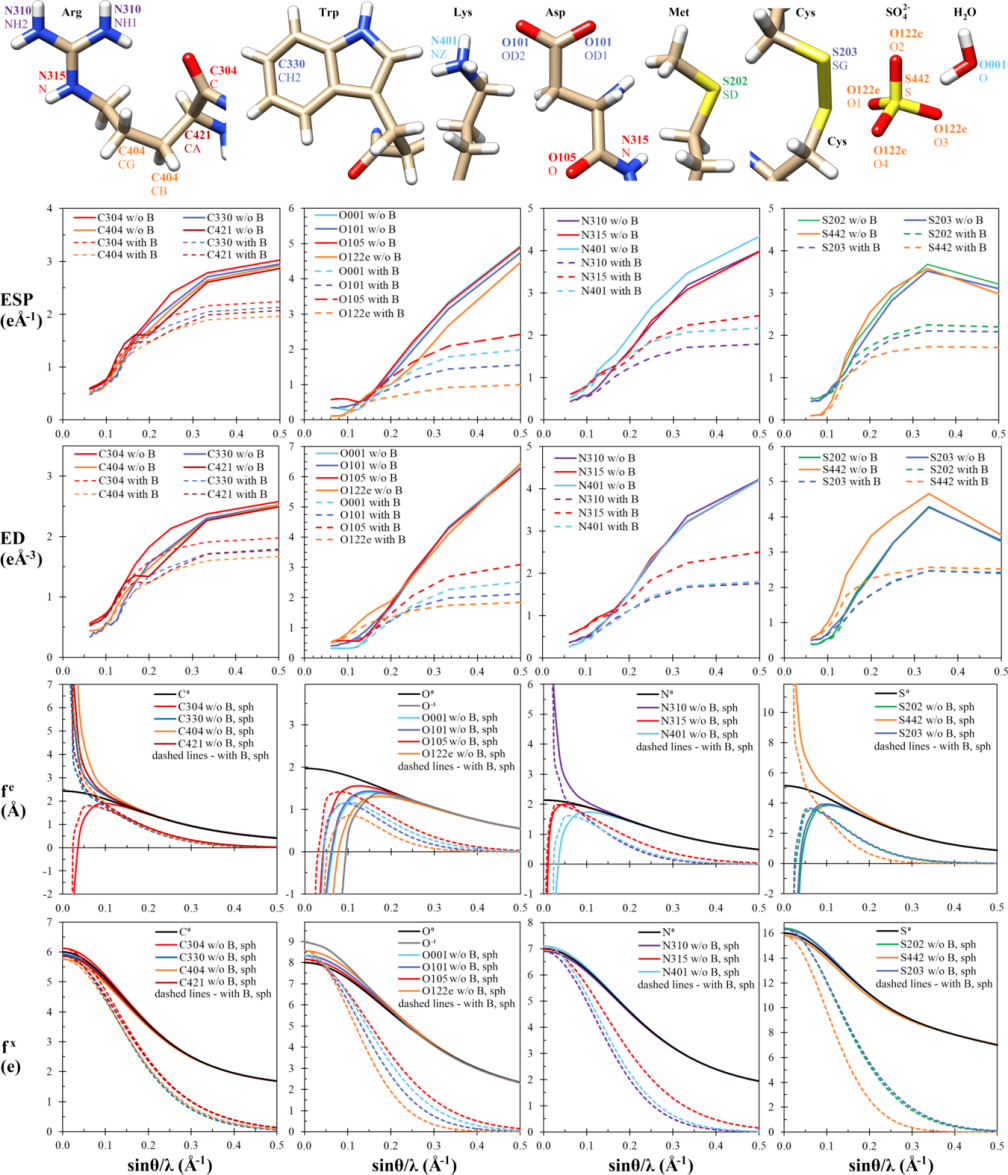
Effect of varying resolution on modeling of chosen atom types. Top panel: atomic structures of atom-type representantives. Each atom-type name (bold font) is assigned together with the atom name in the PDB RCSB database (regular font). Nitrogen, carbon, oxygen, sulfur and hydrogen atoms are shown in blue, beige, red, yellow and white, respectively. Lower panels: the graphs of average values per Å^3^ around atom positions of electrostatic potential (ESP) and electron density (ED), measured in the unscaled maps of lysozyme and proteinase K, atomic scattering factors for electron scattering (*f*^e^) and X-ray scattering (*f*^X^). Scattering factors are calculated for the spherical part of the electron density and protons (sph).

Up to this point we have presented the results for the Fourier maps generated at 1 Å to 4 Å resolution for selected individual amino-acid residues taken from the lysozyme crystal structure. To draw more general conclusions regarding the quantitative difference between TAAM and IAM maps, we computed the values of electrostatic potential and electron density maps at the positions of those atoms at a wider resolution range: up to 8 Å. Moreover, we focused on many more atoms from various functional groups present in the lysozyme and proteinase K crystal structures. The studied atom types included the atoms from functional groups frequently appearing in proteins, depicted in the top panel of Fig. 6 ▸. The atom types present in water molecules and in sulfate ions were also taken into account. TAAM, as opposed to IAM, differentiates atoms with regard to their chemical environment. Thus, in IAM there is only one atomic scattering factor assigned to all atoms of a given chemical element, for example to all carbon atoms. In TAAM there are multiple carbon types with individual atomic scattering factors, for example C421 in the UBDB/MATTS data bank represents the Cα atom, whereas C404 in most cases represents both Cβ and Cγ atoms, as depicted in the top panel of Fig. 6 ▸.

All 3D Fourier electrostatic potential and electron density maps in this study were generated using a 0.3 Å voxel size. To increase the accuracy of the measurement of the map values corresponding to each atom, we decided not to sample the map values simply at the atom positions. Instead, we analyzed the average value of the voxels surrounding every atom. The volume of the surrounding voxels was defined by the covalent radius specific for each element. For instance, the covalent radius of carbon atoms was defined as 0.8 Å (see Table S1). Subsequently, to determine the electrostatic potential and electron density values characteristic for the chosen atom types, the obtained values were further averaged over all atoms belonging to the given atom type present in the lyzosyme or proteinase K crystal structures, and are shown in the graphs with respect to the map resolutions in Figs. 6 ▸ and S7.

Additionally, we investigated how the average values of electrostatic potential and electron density derived from the 3D Fourier maps relate to the electron and X-ray atomic scattering factors embedded in the methods used to generate these maps. Thus, for each atom type, we show the graphs of atomic scattering factors for electron scattering (*f*^e^) and X-ray scattering (*f*^X^), plotted up to the *d* = 1 Å resolution (Figs. 6 ▸ and S7). These graphs are generated for the spherical part of each atom type multipolar expansion.

First, let us investigate the general trends in the dependence of the atomic scattering factors on resolution. The atomic scattering factors for X-ray scattering are always positive, with graphs showing smooth, curved lines and maximum values equal to the sum of the populations of valence and core electrons (*P*_val_ + *P*_core_), as detailed in Table S2. At high scattering angles, the X-ray atomic scattering factors approach zero. The electron atomic scattering factors display similar features to the X-ray atomic scattering factors only for neutral atoms. For charged atoms, the electron scattering factors approaching low scattering angles [low values of 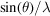] display a sudden slope change and tend towards infinitely high or infinitely low values at 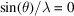.

In all atomic scattering factor graphs, the solid lines represent the values calculated without the thermal smearing effects, while the dashed lines take these effects into account. As expected, thermal smearing decreases the values of the scattering factors, in particular at high scattering angles. There is only one exception to this pattern: if the electron scattering factors decrease to reach negative values at low scattering angles, then the thermal smearing effects tend to increase the electron scattering factor values. This effect is clearly visible in Table S7. It is noteworthy that the influence of thermal smearing effects on the scattering factors is often more pronounced than the influence of the atom type. Also, the averaged experimental *B*-factor values, gathered in Table S3, do not correlate with the *P*_val_ + *P*_core_ values.

Since the atomic scattering factors provide the basis for the generated electrostatic potential and electron density maps, one would expect to find similar general trends for Fourier map values at atomic positions. Indeed, the average electron densities derived from the Fourier maps are always positive. However, at none of the investigated resolutions do the average values of electrostatic potential in the Fourier maps become negative, despite the fact that some of the atomic scattering factors have negative amplitudes in the probed ranges of resolutions. As the resolution increases, the average map values around atoms in electrostatic potential and electron density maps also increase up to a certain point, dependent on the chemical element. In general, two major changes in the slopes are observed in all the electrostatic potential and electron density graphs: at 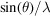 equal to 1/3 and 1/10, which correspond to 1.5 Å and 5 Å resolution, respectively. The dashed lines representing the values taking into account the thermal smearing effects and the solid lines omitting these effects are visibly separated at high (good) resolution region, which underlines the strong effect of thermal smearing on high-resolution maps. On the other hand, the low-resolution maps are practically unaffected by thermal smearing.

At a glance, there is no simple correlation between the atomic scattering factors and the average values of electrostatic potential and electron density for the atoms derived from 3D Fourier maps. The top-left electrostatic potential graph and the related electron density graph in Fig. 6 ▸ depict the average map values for four carbon-atom types of different character. They all follow a similar trend but a few exceptions can be observed. Firstly, C304 – the carbon belonging to the peptide bond – shows the highest map values throughout all resolutions in electrostatic potential and electron density, both with and without thermal smearing, except for a narrow range between 3 Å and 5 Å resolution in the electrostatic potential, where an unusual peak is observed for the C421 Cα atom. On the other hand, the lowest values are noted for C404, which is a member of the nonpolar part of many amino-acid side chains, and at worse resolution for C330 from the aromatic ring. At the same time, the values of the atomic scattering factors for electrons (*f*^e^) are the lowest (also negative) for C304 and the highest for C404. Exactly the opposite situation is observed for *f*^X^, with C304 having the highest values and C404 the lowest. This is in line with the fact that all carbon-atom types, except for C304, carry a positive charge (Table S2).

The order of the lines in the electrostatic potential graph for nitrogen atoms is completely different for the cases with and without thermal smearing effects and it is also different from the order in the electron density graph. N401 shows the highest values in the electrostatic potential maps without taking into account the *B * factors, while including the *B* factors in the calculations strongly affects the lysyl side chains and diminishes the electrostatic potential densities at high spatial frequencies. Among the analyzed nitrogens, only one atom type – N310, representing the two reactive nitrogens in the guanidino group in Arg – displays only positive *f*^e^ values. The other two nitrogens show negative *f*^e^ values at very low spatial frequencies and they carry a negative charge, as visible in the *P*_val_ values in Table S2.

By looking at the electrostatic potential, electron density, *f*^e^ and *f*^X^ graphs for oxygen atoms, both with and without thermal smearing effects, one may think that the oxygen-atom types are remarkably similar in their chemical character. Indeed, small deviations from the general trends are observed only for the O122e atom type, present in sulfate ion, as it features the lowest average electrostatic potential values among other oxygens except for in the 3 Å to 5 Å resolution region. A similar local increase in this region is visible in electron density values. A similar character of all of the studied oxygen-atom types emerges from their negative electron scattering factors. If we arrange all the spatial frequency values at which the electron scattering factors of atom types are equal to zero in a descending order, then the oxygen-atom types would be at the top of that list – see Table S7.

The electrostatic potential and electron density graphs for sulfur atoms have one distinct feature: a negative slope in the high spatial frequencies region, while all the other elements discussed so far feature a positive slope in this region. Also, the S442 sulfur type present in sulfate ions stands out, often bearing the lowest values in electrostatic potential, the highest values in electron density maps and positive *f*^e^ values in the low-resolution region.

We have also analyzed the map values around the positions of several hydrogen atoms of different character. The average values in electrostatic potential and electron density graphs obtained for hydrogen atoms in Fig. S7 are very low and show irregular trends in the resolution domain. Only one analyzed hydrogen-atom type – H103, the Hα atom – features negative values of electron scattering factors in the low-resolution region. However, two hydrogen-atom types, H103 and H108, display a local minimum in the 2.5–3 Å resolution region in both electrostatic potential and electron density graphs.

Focusing on the 3D Fourier maps generated at 1 Å resolution, the average values integrated within the covalent radius distance around atom positions were measured and are shown in Tables S8 and S9 for the average integrated values of electrostatic potential and Tables S10 and S11 for the average integrated values of electron density. The dependence of these average integrated values on the number of electrons of each atom type (*P*_val_ + *P*_core_) is depicted in Fig. S8. Several trends were observed. Firstly, the integrated map values increase with the period of the periodic table of elements, which can be schematically illustrated as (H) < (C, N, O) < (S). Secondly, within the same period of the periodic table, the integrated values increase with the atomic number only in the case of the average integrated values of electron density calculated without thermal smearing effects. In all other cases, the integrated values decrease with the atomic number as far as the relations within the same period are concerned. Thirdly, in most cases, the subtle influence of the populations of electrons (*P*_val_ + *P*_core_) is visible for each element. For carbon atoms, the average integrated values in all maps appear to increase with the number of electrons. For oxygen atoms, the average integrated values appear to decrease with the number of electrons, except for the average integrated values of electron density calculated without thermal smearing effects. For sulfur atoms, the average integrated values of electrostatic potential appear to increase with the number of electrons, while the average integrated values of electron density appear to decrease with the number of electrons. Finally, the graph demonstrates that thermal smearing effects tend to decrease all average integrated map values. Moreover, the thermal smearing effects affect carbon and oxygen atoms to a larger extent than the number of electrons (charge).

In order to draw more general conclusions on differences between TAAM and IAM, an additional analysis for lysozyme was perfomed where the average values of electrostatic potential and electron density derived from the 3D Fourier maps of TAAM and IAM were compared. Tables S12 and S13 show the relative change of the size of the absolute TAAM − IAM difference compared with the TAAM reference value for the average values of electrostatic potential and electron density, respectively. As the resolution decreases (worsens), the differences between TAAM and IAM become more pronounced in the case of electrostatic potential. The largest negative differences were observed for oxygen-atom types, in particular O101 (Table S12). All carbon-atom types and two nitrogen-atom types (N315 and N310) were also characterized with large negative differences. A large positive difference was observed for the N401 atom type. Hydrogen-atom types also point to several disparate TAAM − IAM differences, but here the interpretation of the data should be treated with caution due to very low values measured for the hydrogen atoms in the maps. The impact of thermal smearing was found to be insignificant. In the case of electron density, the TAAM − IAM relative change is small, mostly positive, and more pronounced at higher resolutions, while at a resolution of 8 Å differences between TAAM and IAM were often not detected (Table S13). The results for hydrogen-atom types are unreliable.

To relate our results to the 3D ED experimental data, we have modeled the lysozyme structure with an atomic model of the bulk solvent. The bulk solvent filled the entire unit cell (Fig. S9). Visually, the presence of bulk solvent affects the 3D Fourier maps to a low extent, as shown in Fig. S10. The characteristic effects observed at the charged residue positions at 4 Å resolution in TAAM and IAM maps are indentical, as in the corresponding theoretical maps generated for lysozyme without the addition of bulk solvent. Namely, the eTAAM map contour encompasses less volume of positive electrostatic potential around the negatively charged Asp101 than eIAM, whereas an opposite trend is observed for the positively charged Lys96. If we truncate the data, removing data with a resolution worse than 8 Å (Fig. S11), the differences between TAAM and IAM maps again become negligible, as was the case for lysozyme without bulk solvent. Both 3D Fourier maps are very similar to those generated for the structure without the bulk solvent. We also calculated a Wilson plot (Fig. S12) to relate the values of experimental and theoretical structure factors at various resolutions. The addition of bulk solvent significantly lowers the values of the low-resolution theoretical squared structure factors in the range from *ca* 10 Å to 4 Å. The structure factors corresponding to resolutions better than 4 Å are either not affected or show a small increase in value. Only minor variations in structure-factor values were observed when the set of artificially generated reflection data was replaced with experimentally measured reflection data of less than 100% completeness. We also investigated the impact of scaling the charge of all water molecules in the system to −8 e (see the *Methods*[Sec sec2] section) on the structure factors (Fig. S13). In this case, only a subtle increase in structure-factor values is introduced at *ca* 10 Å resolution. If we compare the structure factors for TAAM and IAM calculated for lysozyme with bulk solvent, with applied charge scaling for water molecules, using experimental reflection data, and an applied scale factor, then TAAM is visibly more in line with the experimental observations than IAM (Fig. S14).

## 4. Discussion

### 4.1. Influence of charges at low resolution

As demonstrated in our previous work (Kulik *et al.*, 2022 ▸), the Fourier electrostatic potential maps calculated with the multipolar approach (eTAAM) at 1.8 Å resolution correspond better to the experimental 3D ED maps at this resolution than the maps calculated with eIAM. Moreover, eTAAM maps show higher sensitivity to charged amino acids than the eIAM maps. Here, we showed that this sensitivity of eTAAM is even more prominent in maps of lower resolutions. The positive electrostatic potential of the negatively charged Asp (and Glu) side chains in eTAAM maps disappear and that of the positively charged Lys and Arg side chains is enhanced when lowering the resolution (Figs. 1 ▸ and 3 ▸). Oxygen atoms from the carboxylate group exhibit map values that are lower by more than 30% (Tables S4 and S12) at 3 Å resolution and almost disappear (*ca* 60 to 80% smaller values) at 4 Å resolution when compared with the charge-ignorant eIAM maps. In fact, oxygen atoms from the carboxylate group are the most sensitive to proper modeling of their charge, especially at lower resolutions, compared with atoms from all the other functional groups studied. For the nitrogen atoms from the ammonium group, electrostatic potential values are enhanced in the Fourier eTAAM maps by *ca* 15% at 3 Å resolution and *ca* 30% at 4 Å resolution (Table S12). The effect is a factor of two smaller than for the oxygen atoms discussed above, most probably because the positive charge in the ammonium groups is delocalized over the hydrogen atoms.

Charge-related information is primarily contained within low-resolution-range reflections. The removal of low-resolution reflections significantly reduces the differences between eTAAM and eIAM maps near charged groups, which is especially visible in the vicinity of carboxylate groups. Thus, it is of utmost importance to properly model and analyze the low-resolution region when dealing with experimental data, both during the data reduction stage and while constructing the solvent model.

The effect of charge also concerns atoms from formally neutral functional groups, though it is more subtle. Valence electrons are redistributed within the molecules due to chemical bonding and other interatomic interactions, leading to partial charges and higher-order multipole moments localized on all atoms. The effect is visible in the Fourier eTAAM − eIAM difference maps, even at 4 Å resolution (Fig. 3 ▸), is less resolution dependent and usually leads to map values at atom positions that are reduced by *ca* 5 to 15% (Table S12).

The relation between the spherical part of the atomic scattering factors for individual atoms and the average electrostatic potential around atoms in the 3D Fourier maps is not straightforward. It is inherently impeded either by the strong influence of the electrostatic potential from the neighboring atoms or by the contribution of the higher-order multipole moments of the atomic scattering factors. Nevertheless, the factors impeding this relation are comparable in their magnitude to the effect of changing the unit charge of an atom.

Additionally, the electrostatic potential maps are affected even by very weak interactions. It has previously been shown that hydrogen bonds are able to modify the electrostatic potential (Chang *et al.*, 1999 ▸). Here, we demonstrated that electrostatic potential values at carboxylate oxygen atoms might change by *ca* 10% (Tables S4 and S5) due to the presence of hydrogen bonding. The electrostatic potential maps are also sensitive to the long-range electrostatic potential generated by the anions appearing in the vicinity. The effects from the hydrogen bonds and other long-range electrostatic interactions are visible at resolutions as low as 4 Å.

The charge effects, and their dependence on resolution, are not as spectacular as could be concluded from the atomic scattering factors computed for atoms having formal charge or even when partial charges are considered (Fig. 6 ▸). This is because the way in which valence electrons are redistributed within the molecules is more sophisticated, and to assess the map values at the position of a particular atom the influence of higher-order multipole moments, contributions from the covalently bonded neighbors and any other long-range interactions have to be taken into account.

### 4.2. Negative values in electrostatic potential maps

Even though there have been many attempts to standardize the processing and validation of single-particle cryo-EM maps (Afonine *et al.*, 2018 ▸; Ortiz *et al.*, 2020 ▸; Lawson *et al.*, 2021 ▸), deposited maps are far from equivalent. Sometimes they are normalized before deposition so that the mean value of the voxels is zero. Sometimes the solvent density is treated as zero. Also, many programs, such as *EMReady* (He *et al.*, 2023 ▸), truncate the negative values for the map density before further processing. Therefore, it is always uncertain what the true meaning of the negative values in deposited maps is. In theory, the negative electron scattering factors from negatively charged oxygen atoms from sulfate anions or carboxylate groups (atom types O122e and O101) without taking into account thermal smearing effects should give a purely negative signal in the electrostatic potential maps at resolutions worse than 6.4 Å and 7.8 Å, respectively, as shown in Table S7. This is in line with expectations for negatively charged oxygen atoms, which give a purely negative signal in the maps at resolutions worse than 9 Å (Jha *et al.*, 2021 ▸). However, our analysis suggests that in the protein environment we rarely see the negative electrostatic potential within the covalent radius distance from the atomic positions in the theoretically generated Fourier maps. The nitrogen atoms, such as the N315 and N401 atom types, also display negative scattering factors within the eTAAM model at resolutions worse than *ca* 15 Å (Table S7) but their positive electrostatic potential values are not diminished. It is worth noting that, in contrast to electrostatic potential and electron density values computed within the protein structures, electron and X-ray scattering factors plotted in Fig. 6 ▸ do not take into account higher-order multipole moments of the atom types, nor do they include contributions from neighboring atoms, the protein environment in general. In the protein environment, negative charges of both these nitrogen atoms are compensated by the positively charged hydrogen atoms H105 and H108. Additionally, one should take into account that due to Fourier summation characteristics, negative values may appear in the Fourier maps due to Fourier series truncation errors (Fourier ripples).

### 4.3. Comparisons with electron density maps

In general, subtle changes in the redistribution of electrons within molecules are much more visible in the electrostatic potential maps than in the electron density maps. Such charge effects are of the order of *ca* 3 to 5% for the Fourier electron density maps at 1 Å resolution and depend less on the resolution than in the case of electrostatic potential maps. The effects observed for hydrogen atoms in electron density maps appear to be a bit greater, but their reliability is limited due to a signal that is weaker than the background signal from the non-hydrogen atoms.

The primary cause of the different behavior of electrostatic potential compared with electron density is the almost complete cancelation of scattering from the positively charged nucleus and the negatively charged electrons. Consequently, even minor variations in electron density result in significant changes in scattering (Zheng *et al.*, 2005 ▸). Another reason is the long-range behavior of electrostatic potential, which is especially visible when lowering the resolution (Wang, 2017 ▸). The electron density is more localized, and the major changes due to electron redistributions occur closer to nuclei. These are the reasons why the Fourier electron density maps are easier to interpret than the electrostatic potential maps, even using simple scattering models such as the IAM.

The correlation between the average values of electrostatic potential and electron density integrated within the covalent radius distance around atom positions and the populations of valence and core electrons for atom types (Fig. S8) are only partially in agreement with the predictions stated by Bochtler (2024 ▸). Generally, the integrated map values increase with the period of the periodic table of elements as (H) < (C, N, O) < (S) and within the same period of the periodic table they increase with the atomic number in the case of the electron density (C < N < O) and they decrease with the atomic number in the case of the electrostatic potential (C > N > O). However, our findings indicate that this statement is true only if we take into account the chemical elements as such, and we ignore more subtle differences between the atom types and the influence of thermal smearing. If we take thermal smearing effects into account, then these predictions are only in agreement for the electrostatic potential 3D Fourier maps. In the case of electron density 3D Fourier maps, a different trend is observed: the integrated values decrease with the atomic number as far as the relations within the same period are concerned. We speculate that the reason behind this trend is the use of IAM in the procedure for *B*-factor refinement using electron diffraction data.

### 4.4. Different *B* factors at different resolutions

On the one hand, the Fourier electrostatic potential or electron density maps are physically meaningless when deprived of thermal smearing effects. On the other hand, it is expected that the *B*-factor values would increase as the resolution of the experimental structure decreases. In this work, the same set of *B* factors was included in the calculations at both low and high resolutions, as the experimental *B* factors for each resolution for the structures with exactly the same atomic coordinates were unavailable. The experimental *B* factors were determined at 1.8 Å resolution for lysozyme and at 1.75 Å resolution for proteinase K, thus we might anticipate that the effect of thermal smearing effects at 1 Å resolution shown in Fig. 1 ▸ is too large with respect to what should be experimentally observed for a sample measured at 1 Å resolution. Nevertheless, thermal smearing affects those Fourier electrostatic potential maps in such a way that the influence of charged residues on the electrostatic potential maps is visible not only at lower resolutions but also at 1 Å resolution. While the impact of thermal smearing on the Fourier electrostatic potential maps is significant at 1 Å resolution, it is barely noticeable at 4 Å resolution. We hypothesize that even with the experimentally determined *B* factors at 4 Å resolution, which would be bigger than those applied in our theoretical maps, the difference between the electrostatic potential maps with and without thermal smearing would also be very small. We can conclude that at high resolution, the accurate determination of *B*-factor values is crucial for the electrostatic potential maps, while at lower resolution the *B* factors do not play a key role. This observation is in agreement with the FSC calculated for structure factors that shows the impact of thermal smearing in Fig. 5 ▸ in Kulik *et al.* (2022 ▸). That figure clearly demonstrates that while the structure factors at low resolution are practically unaffected by thermal smearing effects, at high resolution the difference between the structure factors with and without the *B* factors becomes large.

### 4.5. Resolvability of atoms at various resolutions

There are many opinions about the features that should be observed at a given resolution. According to Henderson *et al.* (2012 ▸), the helical pitch and bulky side chains should be visible at a resolution better than 4.5 Å. We can confirm this statement for uncharged amino acids. However, our maps at 4 Å resolution indicate that the resolvability of amino-acid positions for negatively charged amino acids is low for eTAAM maps, whereas for positively charged amino acids it is low for eIAM maps. This difference for the charged moieties is observed only in Fourier electrostatic potential maps, as Fourier electron density maps are almost insensitive to charges. The presence of holes inside the aromatic rings of Phe and Tyr at the 2σ contour level was observed by us up to *ca* 2 Å resolution, while at *ca* 3 Å resolution the 2D Fourier maps are clearly showing that the inside of the aromatic ring does not reveal any decrease in electrostatic potential or in electron density.

### 4.6. Resolvability of hydrogens

It is expected that hydrogen atoms in single-particle cryo-EM and electron diffraction studies would become visible at somewhat worse resolution than in X-ray diffraction studies, where they are visible typically only at resolutions close to 1 Å or better due to the limited photon scattering power of the hydrogen’s electron (Yip *et al.*, 2020 ▸). In single-particle cryo-EM and electron diffraction, the hydrogen atoms have a larger scattering cross section compared with X-ray crystallography, as the electron beam is scattered not only by the hydrogen’s electron but also by the hydrogen’s proton. Therefore, it is relatively easier to discern the hydrogen atoms among the non-hydrogen atoms in an electrostatic potential map than in an electron density map. Additional densities that agree with the positions of hydrogen atoms were observed in the experimental sharpened single-particle cryo-EM map at 1.25 Å resolution (Yip *et al.*, 2020 ▸). Here, we indeed observe additional densities for the hydrogen atoms for resolutions better than 1.2 Å but without the possibility to distinguish separate peaks and also only in the maps generated without taking into account thermal smearing. It is worth noting that the electrostatic potential and electron density values for hydrogen atoms in theoretical maps are to a great extent affected by the Fourier ripples from the other, heavier elements in their chemical neighborhood, as visible in Fig. S7, where negative slopes were observed. This fact, together with the lack of properly treated thermal factors, limits the accuracy of the determined resolution ranges at which density peaks for hydrogens appear within this work. Nevertheless, it is worth mentioning that the observed peaks close to the hydrogen-atom positions observed in Fourier maps are not located centrally on the hydrogen nuclei positions. Instead, the maxima are shifted further away from the carbon atoms, which is in line with the simulated profile analysis (Nakane *et al.*, 2020 ▸). Measurements of the hydrogen-atom dislocations in a difference map between the the model omitting hydrogen atoms and the experimental data from single-particle cryo-EM (Maki-Yonekura *et al.*, 2023 ▸) and electron diffraction (Takaba *et al.*, 2023 ▸) confirmed this hydrogen-atom behavior for C—H bonds.

### 4.7. Bulk solvent correction

In macromolecular X-ray crystallography, bulk solvent contributes significantly to low-resolution diffraction data. Without correcting for it, calculated structure factors can be systematically larger than observed ones, especially at resolutions worse than 8 Å (Urzhumtsev & Podjarny, 1995 ▸). Our results suggest that in 3D ED this is also the case. Including the diffraction from disordered solvent regions allows us to improve the agreement between theoretical modeling and experimental observations. We also observed that TAAM-generated structure factors are systematically more consistent with experimental measurements than IAM. Modeling the bulk solvent will be crucial for eventually enabling the experimental determination of the charge density of proteins, employing multipolar refinement based on experimental data. For better alignment with experimental data, accurate modeling of many other effects is also necessary, especially dynamical scattering and radiation damage. Monitoring the latter effect during experiments would allow a clearer understanding of its impact on the maps to be gained. TAAM refinement could be useful for that purpose.

## 5. Conclusions

Usage of TAAM, which takes into account the redistribution of valence electron density due to chemical bonding, allowed us to quantify the sensitivity of the Fourier images of electrostatic potential and electron density of biomacromolecules towards electron charge redistribution and its dependence on map resolution. We discovered that the Fourier maps of protein crystals do contain useful information about valence electrons even at *ca* 4 Å resolution. The information content in the Fourier electron density maps is small, about 3 to 5% at 1 Å resolution, and is very weakly dependent on the resolution worsening, but is still present at 4 Å resolution. The Fourier maps of electrostatic potential, on the other hand, are much more sensitive to electron charge redistribution. The map values for all non-hydrogen atoms were affected, by *ca* 5 to 15% at 1 Å resolution, regardless of whether they come from charged or neutral residues, and the effect rises by several per cent with worsening resolution. The biggest effects are observed for charged groups, but effects of covalent bond formations modeled by higher-order electric multipole moments, and such tiny charge fluctuations as those generated by hydrogen bonding or the long-range influence of electrostatic potential of metal cations were also observed. The most spectacular and easy to spot is the sensitivity to the charge of carboxylate groups of amino acids. Such groups are seen to almost disappear from the Fourier maps of electrostatic potential at resolutions lower than 3 Å.

Our results suggest that electron diffraction is more sensitive to tiny changes in the charge distribution than X-ray diffraction. According to our theoretical simulations, this sensitivity is easily observed at resolutions typical for bio­macromolecular data. It is big enough to assume that it will be visible in experimental data, despite the presence of experimental errors. Moreover, the effect should facilitate reliable detection of the protonation state of amino-acid side chains or nucleobases. While high-resolution structures are required to determine the spatial arrangement of atoms, charge information can be derived at resolutions of 3–4 Å. Thus, while it is still necessary to collect data to the highest possible resolution to build the initial atomic model, the effects of charge should be studied on data truncated to lower resolutions. Special care should be taken to collect low-resolution reflections as accurately as possible.

To improve the interpretation of experimental data – including the detection of charge states, accurate modeling of covalent bonds, enhanced resolvability of hydrogen atoms, and the representation of long-range electrostatic effects arising from hydrogen-bond networks and ions embedded in protein structures – TAAM scattering factors should be employed, particularly for lower-resolution datasets. The practical implementation of TAAM in refinement programs extensively utilized in the field of macromolecular crystallography is underway at present. Such implementation would allow us to perform a large-scale comparison between our theoretically generated electrostatic potential maps and the experimental data, with the focus on capturing multiple effects characteristic for 3D electron diffraction data, such as dynamical scattering, radiation damage, missing reflections or other systematic effects.

## Supplementary Material

Supporting figures and tables. DOI: 10.1107/S2052252525008383/ur5005sup1.pdf

Theoretical 3D Fourier maps of electrostatic potential and electron density generated with TAAM structure factors for lysozyme: https://doi.org/10.18150/7NQCCU

Theoretical 3D Fourier maps of electrostatic potential and electron density generated with TAAM structure factors for proteinase K: https://doi.org/10.18150/K4A12G

Theoretical 3D Fourier maps of electrostatic potential generated with TAAM structure factors for lysozyme with bulk solvent: https://doi.org/10.18150/QJ6U6S
