# Supplementary material for: Protein electrostatic potential Fourier maps calculated using the transferable aspherical atom model and the independent atom model across resolutions
Source: IUCrJ. 2025 Oct 17;12(Pt 6):616–32. doi: 10.1107/S2052252525008383 (PMC12573929; doi:10.1107/S2052252525008383)
Supplement: Supplementary file 1 [file m-12-00616-sup1.pdf]

IUCrJ

Volume 12 (2025)

Supporting information for article:

Protein electrostatic potential Fourier maps calculated using the transferable aspherical atom model and the independent atom model across resolutions

Marta Kulik and Paulina Maria Dominiak

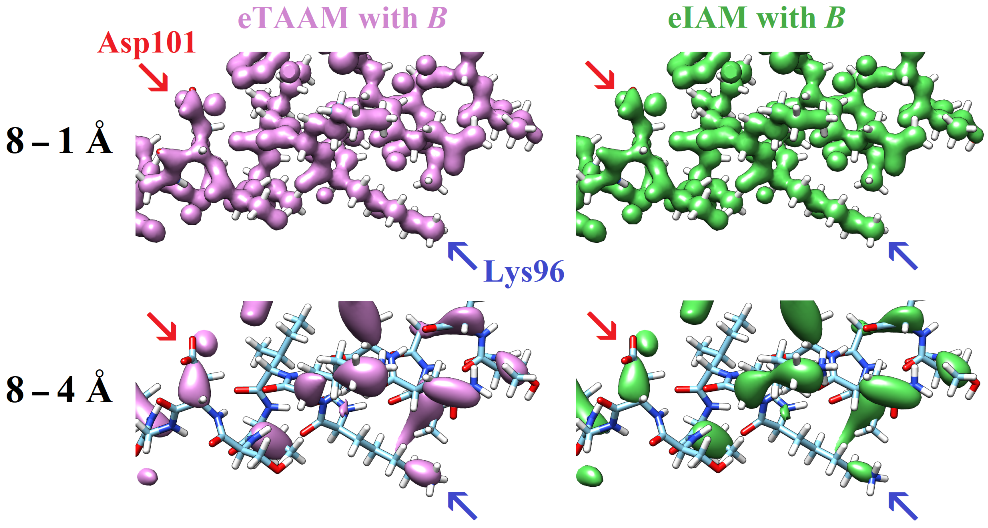

Fig. S1. 3D Fourier electrostatic potential maps of lysozyme calculated using the resolution ranges 8 – 1 Å and 8 – 4 Å with an atomic structure shown for a short helical fragment only. The maps are calculated using TAAM with accounting for B factors, i. e. thermal smearing effects (pink) and using IAM with B factors (light green). Two chosen, oppositely charged amino acids are indicated with arrows. All maps are shown at 2 sigma contour.

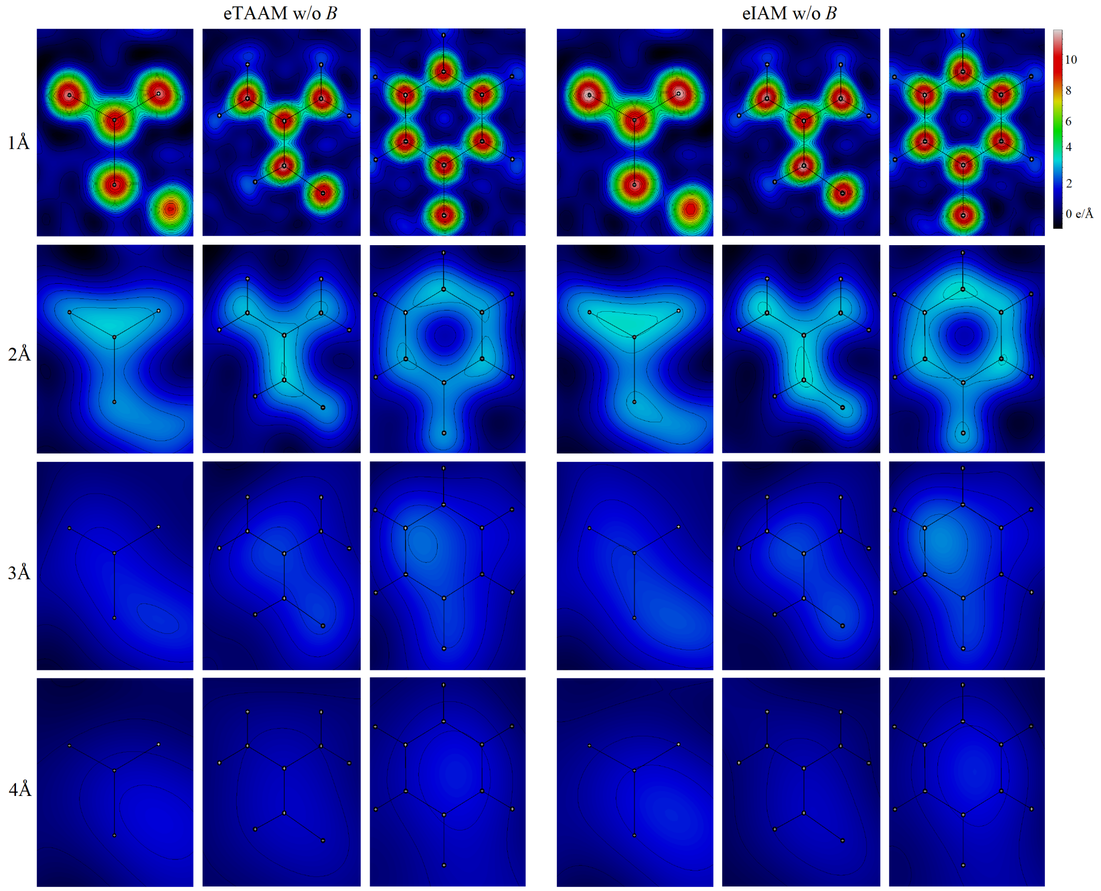

Fig. S2. 2D electrostatic potential density maps of Asp66, Arg14 and Phe3 lysozyme amino acid side chains at resolutions $d_{min} = 1 - 4$ Å. Maps are calculated using eTAAM (left panel) and eIAM (right panel) and are omitting the influence of thermal smearing (w/o B).

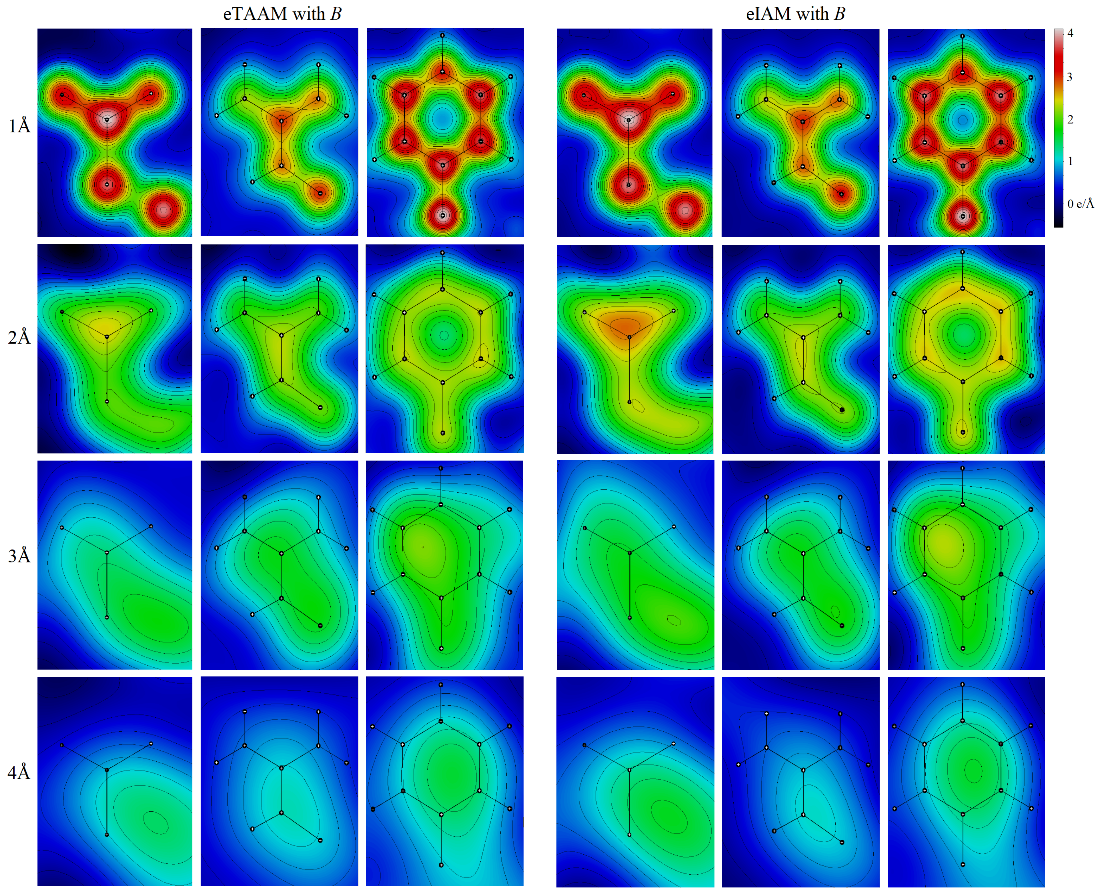

Fig. S3. 2D electrostatic potential density maps of Asp66, Arg14 and Phe3 lysozyme amino acid side chains at resolutions $d_{min} = 1 - 4$ Å. Maps are calculated using eTAAM (left panel) and eIAM (right panel), taking into account the thermal smearing effects (with B).

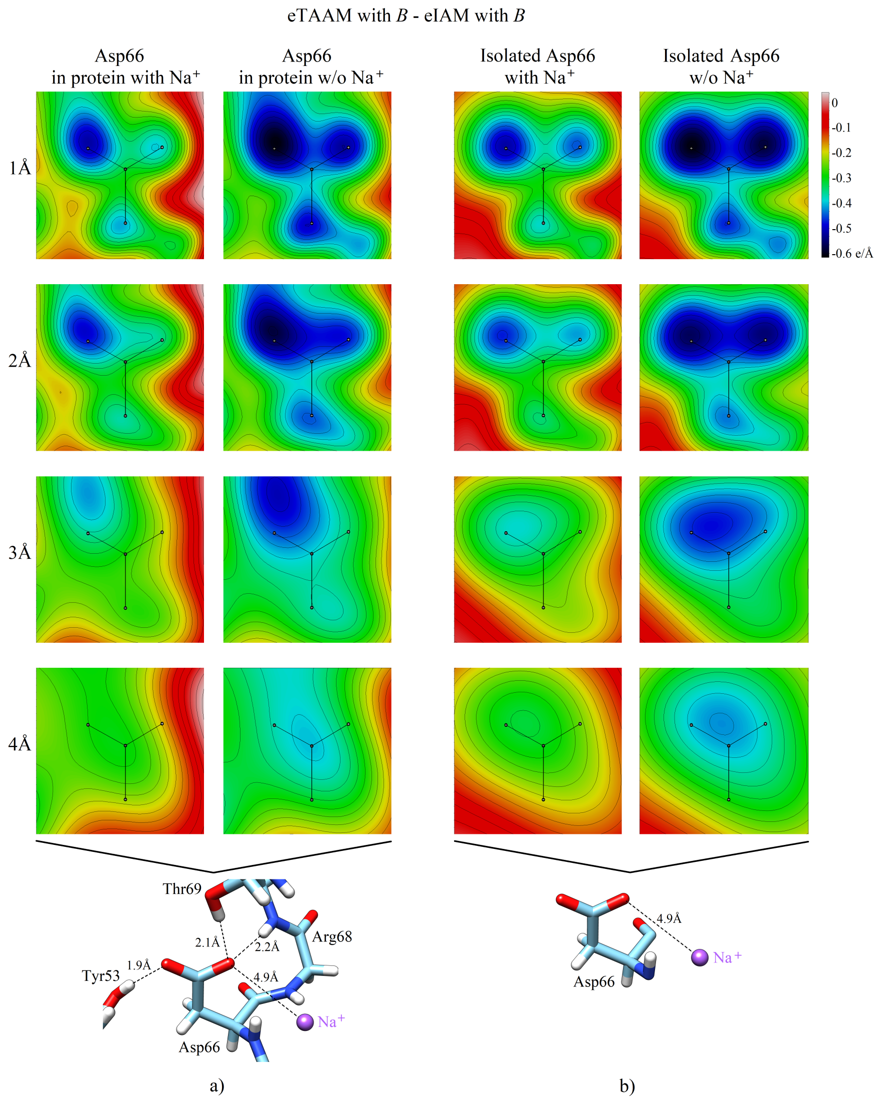

Fig. S4. Influence of hydrogen bond network and Na^+ ion on the electrostatic potential maps of Asp66 lysozyme side chains at resolutions $d_{min} = 1 - 4$ Å. The maps take into account the thermal smearing effects (with B). a) 2D Fourier TAAM – IAM maps for Asp66 calculated in the protein environment with Na^+ (left column) and without Na^+ (right column). The left column directly corresponds to Figure 3b but here the scale was adjusted to the other panels of the current figure for better visibility. The structural view of Asp66, the hydrogen bond network around the carboxylate group and the Na^+ ion are shown in the bottom panel. The distances between oxygen and hydrogen atoms or oxygen and sodium atoms are marked with dashed lines. b) 2D Fourier TAAM – IAM maps for Asp66 calculated without the protein environment with Na^+ (left column) and without Na^+ (right column). The structural views of Asp66 and the Na^+ ion are shown in the bottom panel. The distance between oxygen and sodium atoms is marked with a dashed line.

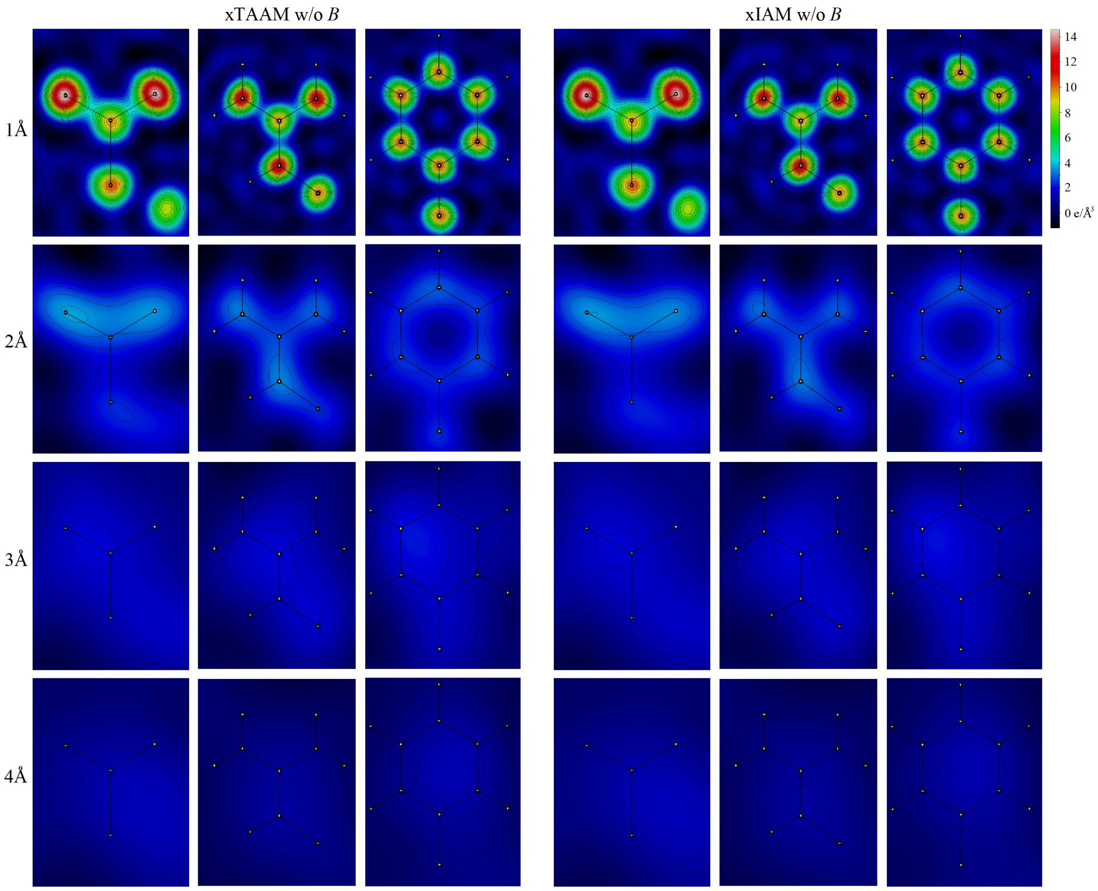

Fig. S5. 2D electron density maps of Asp66, Arg14 and Phe3 lysozyme amino acid side chains at resolutions $d_{min} = 1 - 4$ Å. Maps are calculated using xTAAM (left panel) and xIAM (right panel) and are omitting the influence of thermal smearing (w/o B).

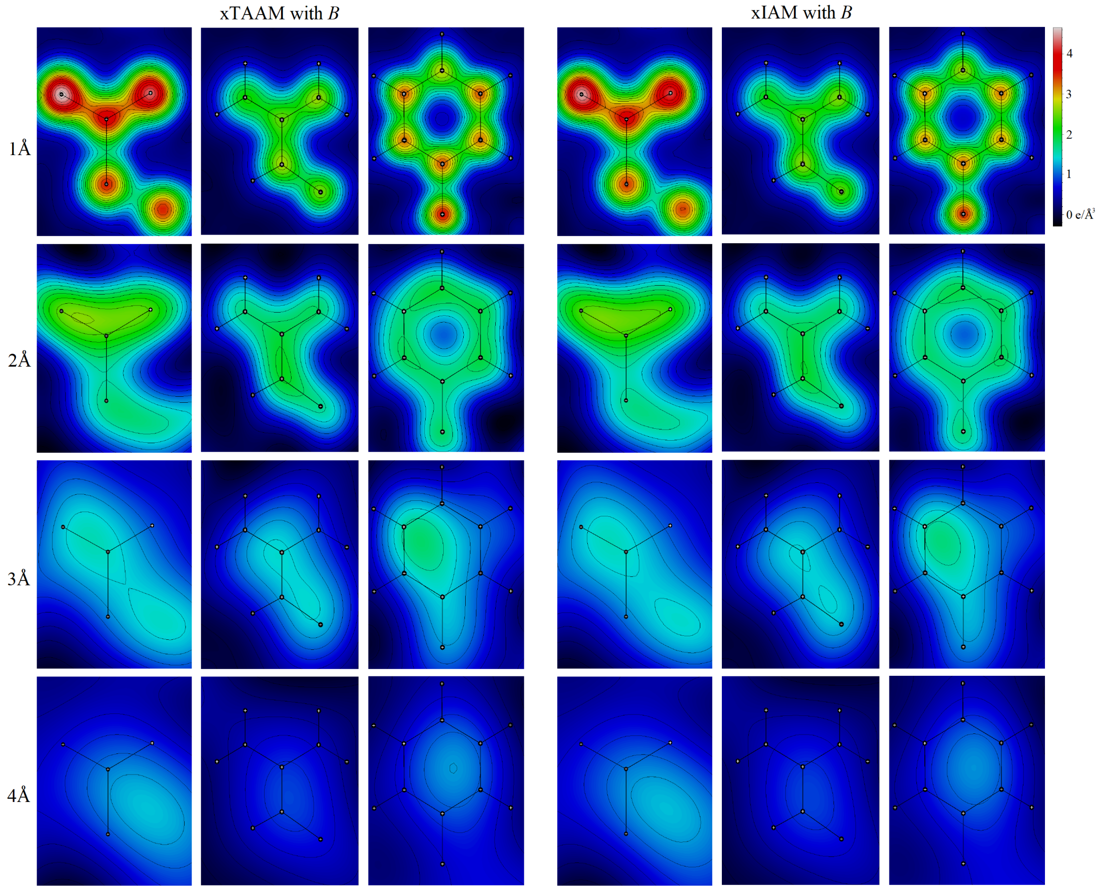

Fig. S6. 2D electron density maps of Asp66, Arg14 and Phe3 lysozyme amino acid side chains at resolutions $d_{min} = 1 - 4$ Å. Maps are calculated using xTAAM (left panel) and xIAM (right panel), taking into account the thermal smearing effects (with B).

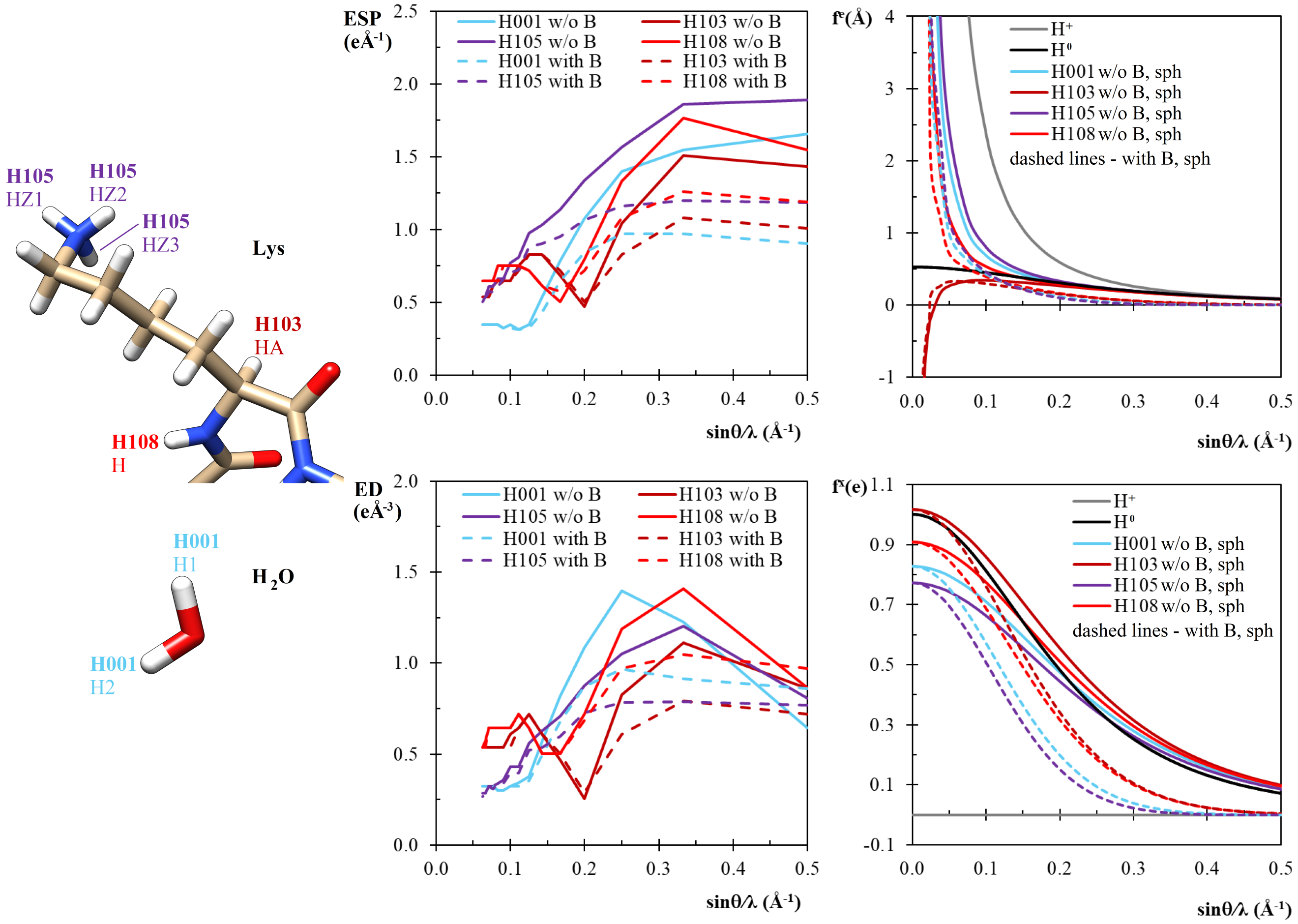

Fig. S7. Effect of varying resolution on hydrogen atom types modeling. Left panel: atomic structures of atom types representants. Each atom type name (bold font) is assigned together with the atom name in PDB RCSB database (regular font). Nitrogen, carbon, oxygen and hydrogen atoms are shown in blue, beige, red and white, respectively. Right panels: the graphs of average values around atom positions of electrostatic potential (ESP) and electron density (ED), measured in the unscaled maps of lysozyme and proteinase K. Atomic scattering factors for electron scattering (f^e) and X-ray scattering (f^X). Scattering factors are calculated for the spherical part of the electron density and protons (sph).

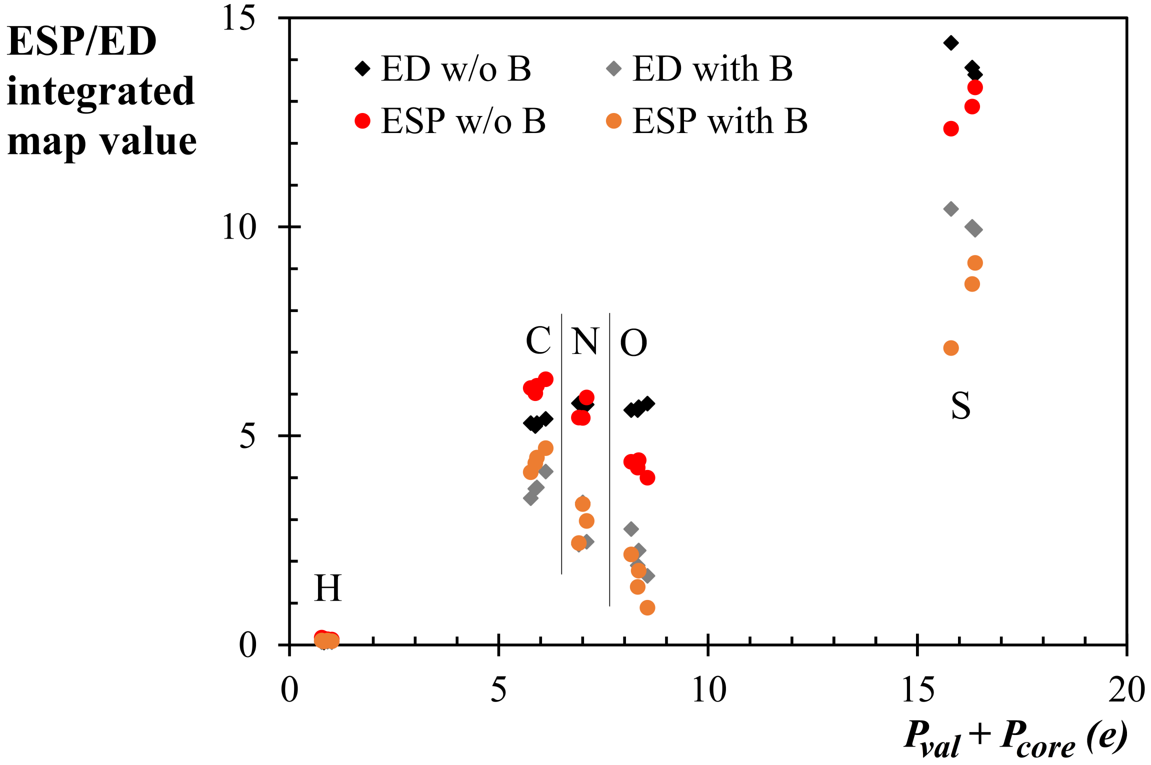

Fig. S8. The correlation between the average values of electrostatic potential (ESP) and electron density (ED) integrated within the covalent radius distance around atom positions and the populations of valence and core electrons for atom types ($P_{val}+P_{core}$). The integrated values were measured in the unscaled maps of lysozyme and proteinase K, calculated at 1 Å resolution, with or without thermal smearing effects. The integrated map values were normalized to resemble a sampling per 1 Å^3. Each group of points corresponds to one chemical element, marked in the graph: H, C, N, O, or S.

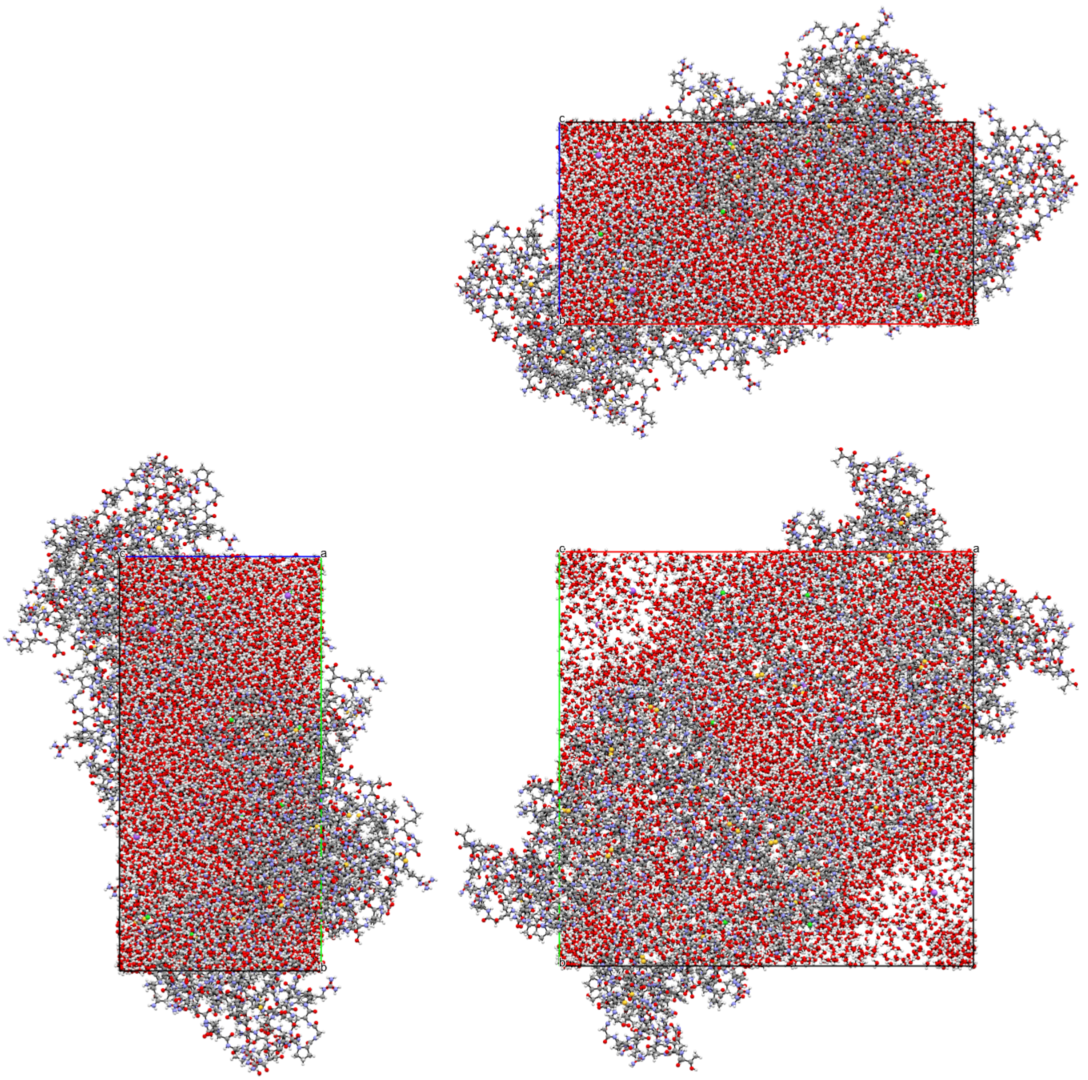

Fig. S9. Views of the lysozyme unit cell with bulk solvent.

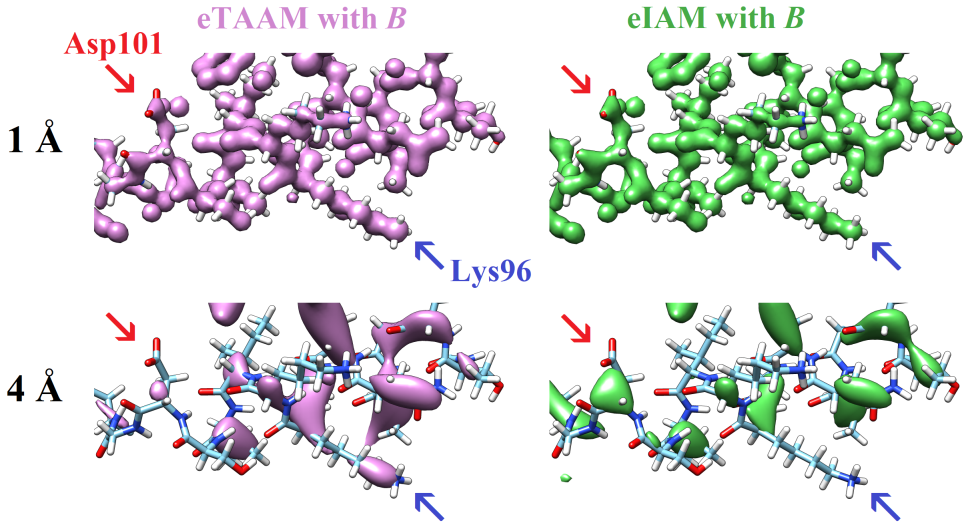

Fig. S10. 3D Fourier electrostatic potential maps of lysozyme with bulk solvent, calculated at resolutions 1 Å and 4 Å with an atomic structure shown for a short helical fragment only. The maps are calculated using TAAM with accounting for B factors, i. e. thermal smearing effects (pink) and using IAM with B factors (light green). Two chosen, oppositely charged amino acids are indicated with arrows. All maps are shown at 2 sigma contour.

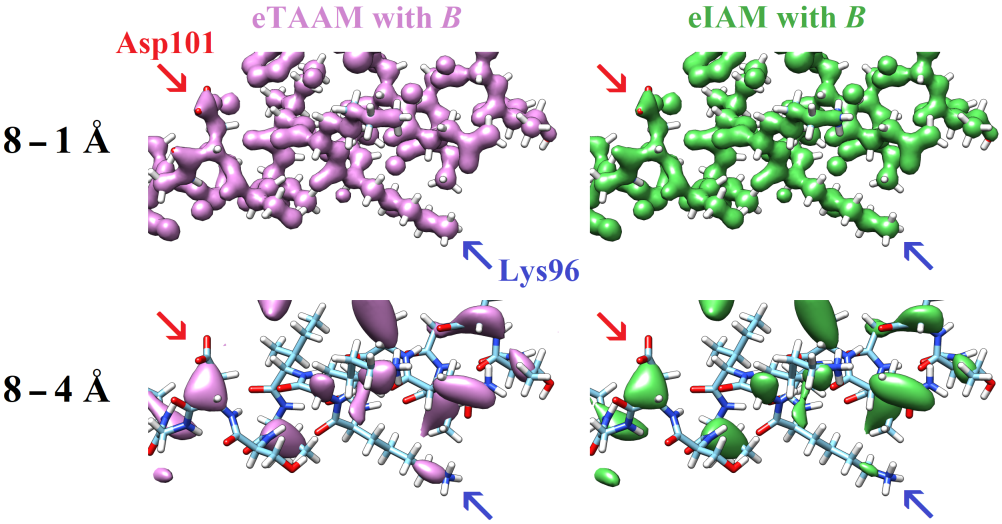

Fig. S11. 3D Fourier electrostatic potential maps of lysozyme with bulk solvent, calculated using the resolution ranges 8 − 1 Å and 8 − 4 Å with an atomic structure shown for a short helical fragment only. The maps are calculated using TAAM with accounting for B factors, i. e. thermal smearing effects (pink) and using IAM with B factors (light green). Two chosen, oppositely charged amino acids are indicated with arrows. All maps are shown at 2 sigma contour.

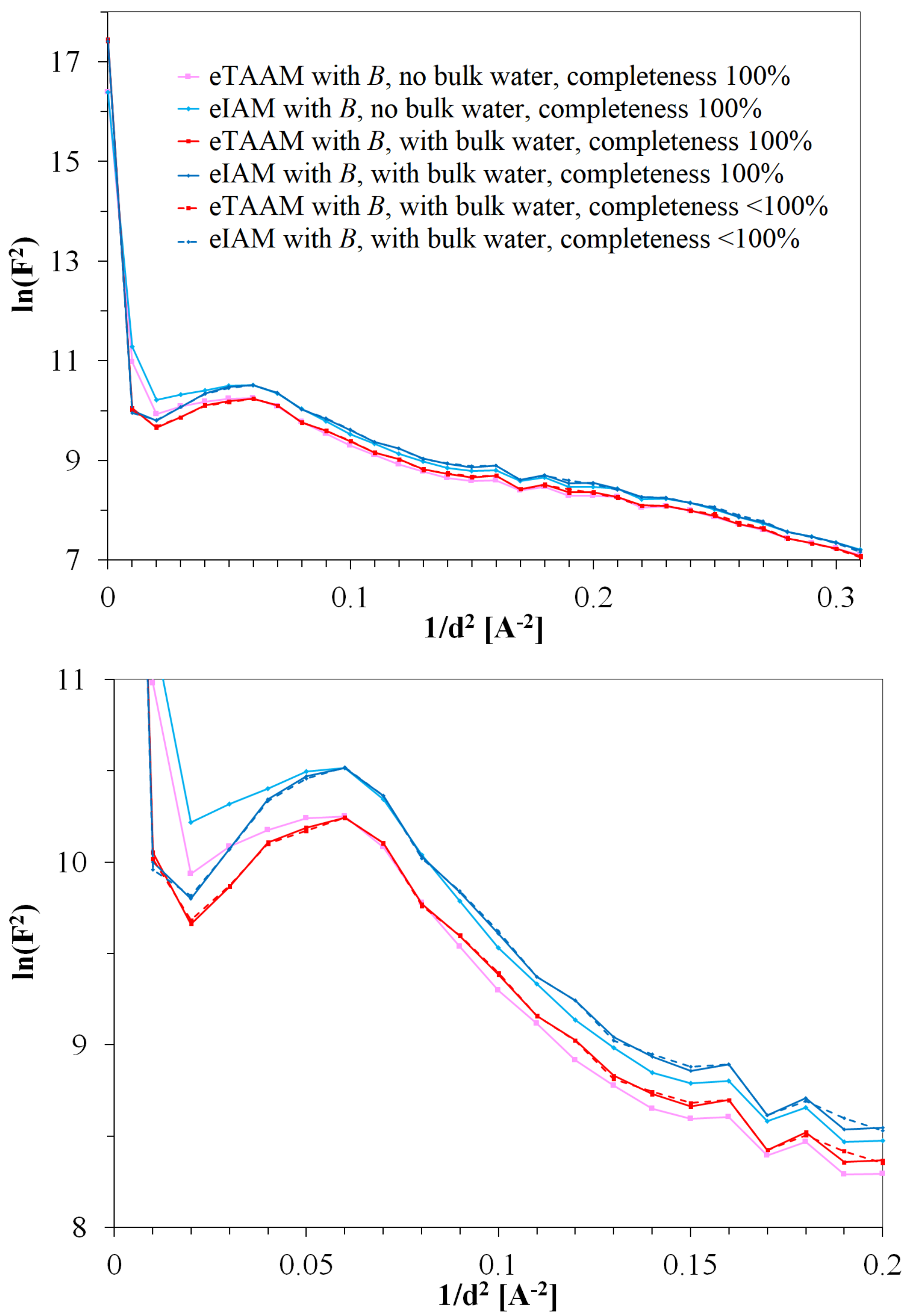

Fig. S12. Wilson plot for electron diffraction structure factors calculated with TAAM and IAM for lysozyme structure without bulk water (as deposited in the PDB RCSB) and for lysozyme structure with additional water molecules (with bulk water). Completeness 100% indicates the usage of artificial reflection indices, whereas completeness <100% indicates the usage of experimental reflection indices. The bottom panel shows a magnification of a chosen fragment of the top graph.

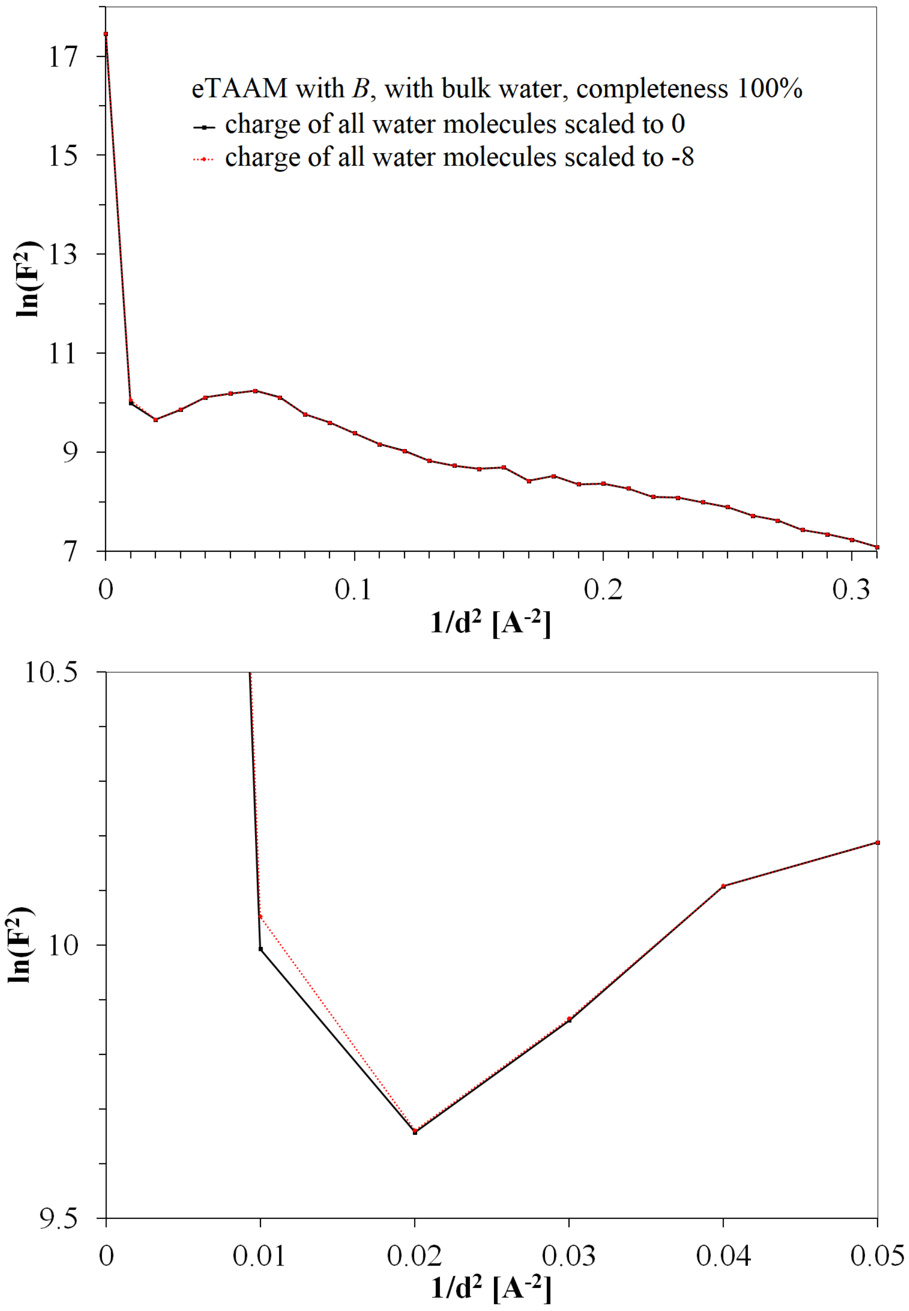

Fig. S13. Wilson plot for electron diffraction structure factor calculated with TAAM for lysozyme structure with additional water molecules (with bulk water), showing the impact of scaling the charge of water molecules. Completeness 100% indicates the usage of artificial reflection indices. The bottom panel shows a magnification of a chosen fragment of the top graph.

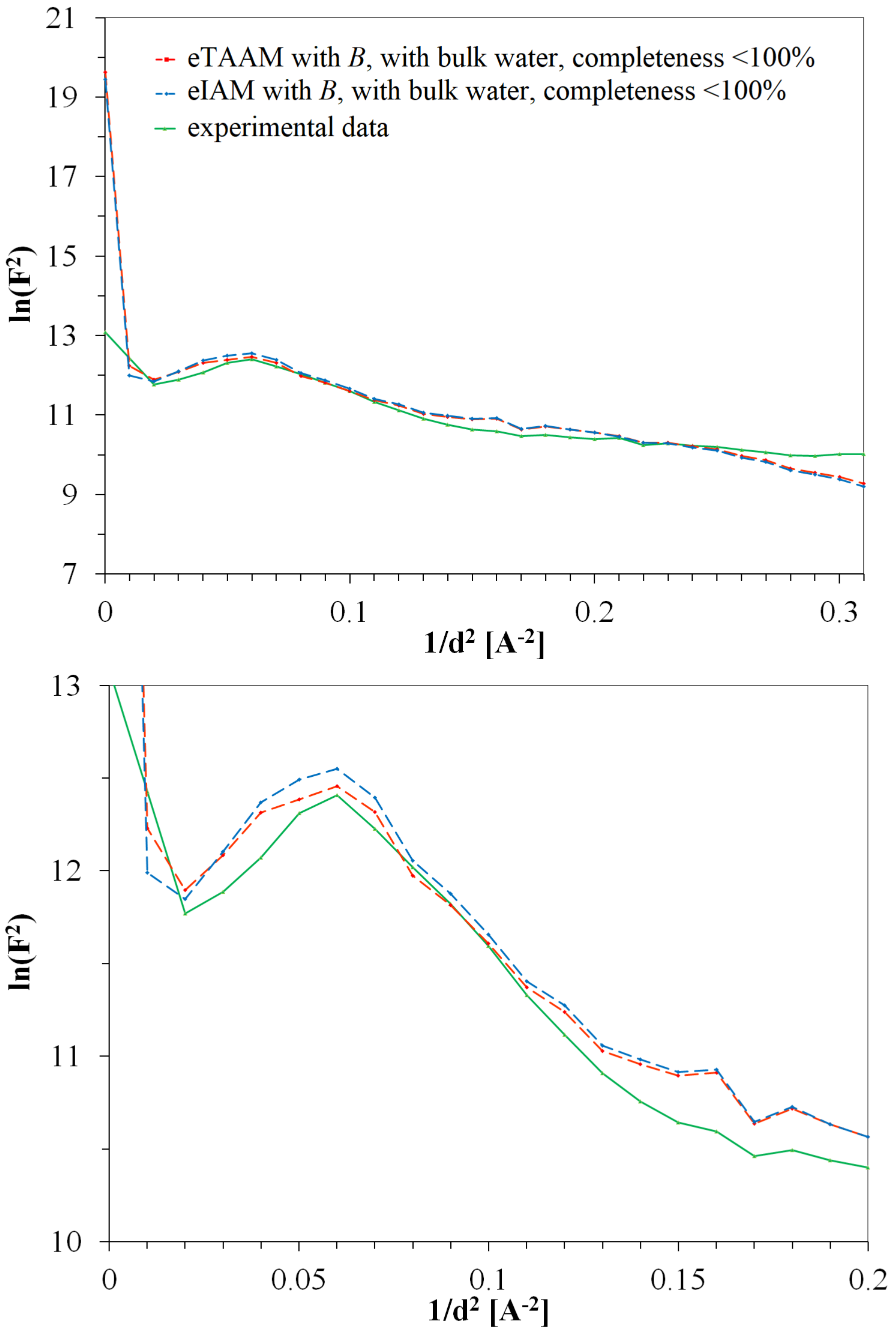

Fig. S14. Wilson plot for electron diffraction structure factors calculated with TAAM and IAM for lysozyme structure with additional water molecules (with bulk water), adjusted by a scale factor to match the experimental data range. Completeness ¡100% indicates the usage of experimental reflection indices. The bottom panel shows a magnification of a chosen fragment of the top graph.

Table S1. *Atom types for which the average values of the density map around atom positions were calculated. The radius around atom position, the encompassed number of grid points sampled and the overall number of sampled atoms in lysozyme and proteinase K are shown for each atom type.*

Atom type	Radius [Å]	Number of grid points	Number of atoms
C304	0.8	2103	408
C330	0.8	2103	7
C404	0.8	2103	42
C421	0.8	2103	408
N310	0.7	1365	44
N315	0.7	1365	430
N401	0.7	1365	14
O001	0.6	895	220
O101	0.6	895	56
O105	0.6	895	408
O122e	0.6	895	8
S202	1.0	4139	7
S203	1.0	4139	12
S442	1.0	4139	2
H001	0.3	93	440
H103	0.3	93	363
H105	0.3	93	42
H108	0.3	93	395

Table S2. *The values of populations of valence and core electrons for atom types shown in the Figure 6 and Figure S7.*

	Pval	Pcore
Cneutr	4.0000	2
C304	4.1240	2
C330	3.9160	2
C404	3.7660	2
C421	3.8720	2
Nneutr	5.0000	2
N310	4.9130	2
N315	5.0090	2
N401	5.1000	2
Oneutr	6.0000	2
O001	6.3440	2
O101	6.3190	2
O105	6.1610	2
O122e	6.5497	2
Sneutr	6.0000	10
S202	6.3820	10
S203	6.3090	10
S442	5.8011	10
Hneutr	1.0000	0
H001	0.8280	0
H103	1.0160	0
H105	0.7730	0
H108	0.9090	0

Table S3. *Average B factor values in experimental structures calculated for atom types shown in the Figure 6 and Figure S7.*

	Average B factor (Å^2)		
Atom type	Lysozyme	Proteinase K	Average for Lysozyme and Proteinase K
C304	11.76	9.23	10.03
C330	15.13	7.90	13.32
C404	13.25	11.75	12.25
C421	11.42	9.55	10.14
N310	24.20	16.83	20.51
N315	12.51	9.07	10.19
N401	17.83	18.01	17.93
O001	14.27	14.63	14.49
O101	18.61	19.53	19.23
O105	12.75	10.36	11.12
O122e	N/A	28.04	28.04
S202	7.60	22.35	18.14
S203	15.21	20.82	17.08
S442	N/A	35.55	35.55
H001	21.41	21.94	21.73
H103	13.53	11.47	12.13
H105	26.75	27.02	26.90
H108	13.46	11.21	11.93

Table S4. *The values of chosen 2D Fourier maps at the left oxygen atom position of Asp66 from lysozyme as shown in the Figures 3, S2 and S3, calculated in the protein environment. The relative change describes the size of the absolute TAAM – IAM change in comparison to the TAAM reference value.*

Resolution (Å)	**eTAAM w/o B – eIAM w/o B (eÅ^{-1})**	**eTAAM with B – eIAM with B (eÅ^{-1})**
1	-0.633	-0.511
2	-0.584	-0.475
3	-0.381	-0.360
4	-0.289	-0.283

Resolution (Å)	**eTAAM w/o B (eÅ^{-1})**	**eTAAM with B (eÅ^{-1})**
1	11.259	3.362
2	2.659	1.748
3	1.061	0.923
4	0.362	0.346

Resolution (Å)	**Relative change (%)**	**Relative change (%)**
1	-6	-15
2	-22	-27
3	-36	-39
4	-80	-82

Table S5. *The values of chosen 2D Fourier maps at the left oxygen atom position of Asp66 from lysozyme as shown in the Figure S4, calculated in the protein environment without Na^+ and for isolated Asp66 with or without Na^+. The relative change describes the size of the absolute TAAM – IAM change in comparison to the TAAM reference value.*

	**eTAAM with B – eIAM with B (eÅ^{-1})**		
Resolution (Å)	Asp66 in protein w/o Na^+	Isolated Asp66 with Na^+	Isolated Asp66 w/o Na^+
1	-0.599	-0.517	-0.605
2	-0.563	-0.467	-0.555
3	-0.448	-0.379	-0.467
4	-0.371	-0.309	-0.397

	**eTAAM with B (eÅ^{-1})**		
Resolution (Å)	Asp66 in protein w/o Na^+	Isolated Asp66 with Na^+	Isolated Asp66 w/o Na^+
1	3.274	3.319	3.231
2	1.668	1.547	1.467
3	0.848	0.842	0.768
4	0.243	0.427	0.324

	**Relative change (%)**		
Resolution (Å)	Asp66 in protein w/o Na^+	Isolated Asp66 with Na^+	Isolated Asp66 w/o Na^+
1	-18	-16	-19
2	-34	-30	-38
3	-53	-45	-61
4	-153	-72	-122

Table S6. *The values of chosen 2D Fourier maps at the left oxygen atom position of Asp66 from lysozyme as shown in the Figures 4, S5 and S6 calculated in the protein environment. The relative change describes the size of the absolute TAAM – IAM change in comparison to the TAAM reference value.*

Resolution (Å)	**xTAAM w/o B – xIAM w/o B (eÅ^{-3})**	**xTAAM with B – xIAM with B (eÅ^{-3})**
1	0.161	0.131
2	0.139	0.092
3	0.042	0.034
4	0.010	0.008

Resolution (Å)	**xTAAM w/o B (eÅ^{-3})**	**xTAAM with B (eÅ^{-3})**
1	14.582	4.654
2	3.625	2.471
3	1.531	1.338
4	0.681	0.650

Resolution (Å)	**Relative change (%)**	**Relative change (%)**
1	1	3
2	4	4
3	3	3
4	1	1

Table S7. *Resolutions at which the electron scattering factors of atom types become negative. The values of* $\frac{sin(\theta)}{\lambda}$ *are taken from the data shown in the Figure 6.*

Atom type	$\frac{sin(\theta)}{\lambda}$ (Å^{-1})	Resolution (Å)
O-1 ion	0.0960	5.2
O122e w/o B	0.0780	6.4
O001 w/o B	0.0640	7.8
O101 w/o B	0.0620	8.1
O122e with B	0.0520	9.6
O001 with B	0.0440	11.4
O105 w/o B	0.0430	11.6
O101 with B	0.0430	11.6
S202 w/o B	0.0400	12.5
S203 w/o B	0.0370	13.5
C304 w/o B	0.0350	14.3
N401 w/o B	0.0340	14.7
O105 with B	0.0300	16.7
S202 with B	0.0280	17.9
S203 with B	0.0250	20.0
C304 with B	0.0240	20.8
N401 with B	0.0240	20.8
N315 w/o B	0.0100	50.0
N315 with B	0.0075	66.7

Table S8. *Average values of electrostatic potential (ESP) integrated within the covalent radius distance around atom positions, measured in the unscaled maps of lysozyme and proteinase K, calculated at 1 Å resolution without thermal smearing effects. The integrated map values were normalized to resemble a sampling per 1 $Å^3$. Their standard deviations are shown.*

Atom type	ESP w/o B (integrated map value)	SD
H103	0.13	0.01
H108	0.14	0.01
H001	0.15	0.02
H105	0.18	0.02
O122e	4.00	0.11
O101	4.24	0.11
O105	4.38	0.10
O001	4.42	0.12
N315	5.43	0.13
N310	5.44	0.11
N401	5.92	0.17
C421	6.02	0.20
C404	6.15	0.24
C330	6.20	0.12
C304	6.36	0.20
S442	12.35	0.54
S203	12.88	0.27
S202	13.34	0.34

Table S9. *Average values of electrostatic potential (ESP) integrated within the covalent radius distance around atom positions, measured in the unscaled maps of lysozyme and proteinase K, calculated at 1 Å resolution with thermal smearing effects. The integrated map values were normalized to resemble a sampling per 1 $Å^3$. Their standard deviations are shown.*

Atom type	ESP with B (integrated map value)	SD
H001	0.08	0.02
H103	0.09	0.01
H105	0.11	0.02
H108	0.11	0.02
O122e	0.89	0.47
O101	1.39	0.38
O001	1.78	0.43
O105	2.17	0.47
N310	2.44	0.60
N401	2.97	0.70
N315	3.37	0.54
C404	4.13	0.77
C421	4.35	0.54
C330	4.48	0.80
C304	4.71	0.58
S442	7.10	5.29
S203	8.63	0.98
S202	9.14	1.41

Table S10. *Average values of electron density (ED) integrated within the covalent radius distance around atom positions, measured in the unscaled maps of lysozyme and proteinase K, calculated at 1 Å resolution without thermal smearing effects. The integrated map values were normalized to resemble a sampling per 1 Å^3. Their standard deviations are shown.*

Atom type	ED w/o B (integrated map value)	SD
H001	0.06	0.01
H105	0.08	0.01
H103	0.08	0.01
H108	0.08	0.01
C421	5.24	0.05
C404	5.31	0.09
C330	5.31	0.08
C304	5.41	0.05
O101	5.62	0.03
O105	5.62	0.04
O001	5.69	0.04
N315	5.74	0.04
N401	5.75	0.03
O122e	5.77	0.02
N310	5.78	0.03
S202	13.64	0.11
S203	13.81	0.10
S442	14.40	0.14

Table S11. *Average values of electron density (ED) integrated within the covalent radius distance around atom positions, measured in the unscaled maps of lysozyme and proteinase K, calculated at 1 Å resolution with thermal smearing effects. The integrated map values were normalized to resemble a sampling per 1 Å^3. Their standard deviations are shown.*

Atom type	ED with B (integrated map value)	SD
H103	0.07	0.01
H105	0.07	0.01
H001	0.08	0.01
H108	0.09	0.01
O122e	1.65	0.52
O101	1.90	0.49
O001	2.26	0.52
N310	2.40	0.67
N401	2.47	0.75
O105	2.77	0.57
N315	3.41	0.56
C404	3.51	0.63
C421	3.74	0.42
C330	3.77	0.68
C304	4.15	0.41
S202	9.93	1.48
S203	10.00	1.31
S442	10.43	5.20

Table S12. *The relative change of the size of the absolute TAAM – IAM change in comparison to the TAAM reference value in 3D Fourier maps of lysozyme, generated with the electron scattering factors without and with thermal smearing effects.*

	Relative change [%]									
	eTAAM w/o B – eIAM w/o B					eTAAM with B – eIAM with B				
Resolution (Å)	1	2	3	4	8	1	2	3	4	8
C304	-7	-8	-11	-13	-13	-8	-9	-11	-13	-13
C330	-8	-9	-8	-3	11	-7	-8	-7	-2	11
C404	-10	-12	-14	-12	-6	-11	-12	-12	-11	-6
C421	-11	-13	-13	-12	-12	-12	-13	-12	-12	-12
N310	-7	-10	-6	-1	4	-7	-7	-4	-1	3
N315	-8	-11	-10	-10	-12	-9	-10	-10	-11	-12
N401	0	3	13	19	32	7	8	15	20	31
O001	-7	-13	-14	-7	0	-9	-12	-14	-7	0
O101	-11	-25	-43	-63	-63	-26	-32	-47	-65	-62
O105	-7	-13	-16	-16	-14	-11	-14	-16	-18	-14
S202	-1	-1	-4	2	7	-1	-1	-3	3	8
S203	-6	-7	-11	-16	-26	-8	-8	-12	-17	-25
H001	0	0	-7	14	0	0	0	0	0	0
H103	-14	-11	-20	-14	-20	-13	-14	0	-14	-20
H105	10	16	23	23	29	17	21	23	29	29
H108	-15	-9	0	-17	0	-11	-13	-25	-17	0

Table S13. *The relative change of the size of the absolute TAAM – IAM change in comparison to the TAAM reference value in 3D Fourier maps of lysozyme, generated with the X-ray scattering factors without and with thermal smearing effects.*

	Relative change [%]									
	xTAAM w/o B – xIAM w/o B					xTAAM with B – xIAM with B				
Resolution (Å)	1	2	3	4	8	1	2	3	4	8
C304	2	2	2	1	0	2	1	1	1	1
C330	5	4	2	1	0	4	3	2	1	-2
C404	4	4	3	1	0	3	3	2	1	0
C421	4	4	2	1	1	3	3	2	1	0
N310	4	4	2	0	0	3	2	1	0	0
N315	4	3	1	1	1	3	2	1	1	0
N401	3	2	-1	-2	0	1	1	-1	-1	-3
O001	2	4	2	0	0	3	3	1	0	0
O101	2	4	3	2	0	3	4	3	3	0
O105	2	3	2	2	0	3	3	1	0	0
S202	1	0	2	1	0	1	1	2	1	0
S203	1	1	1	0	0	1	1	1	1	0
H001	-17	0	0	0	0	0	0	0	0	0
H103	13	14	0	0	0	17	0	0	0	0
H105	-11	-13	-6	0	0	0	0	-13	0	0
H108	0	0	0	0	0	0	0	0	0	0
